# Heteroscedastic Reaction Norm Models Improve the Assessment of Genotype by Environment Interaction for Growth, Reproductive, and Visual Score Traits in Nellore Cattle

**DOI:** 10.3390/ani12192613

**Published:** 2022-09-29

**Authors:** Ivan Carvalho Filho, Delvan A. Silva, Caio S. Teixeira, Thales L. Silva, Lucio F. M. Mota, Lucia G. Albuquerque, Roberto Carvalheiro

**Affiliations:** 1 School of Agricultural and Veterinarian Sciences, São Paulo State University (UNESP), Jaboticabal 14884-900, Brazil; 2 Department of Animal Science, Universidade Federal de Viçosa, Viçosa 36570-000, Brazil; 3 Department of Agronomy, Food, Natural Resources, Animals and Environment (DAFNAE), University of Padua, Legnaro, 35020 Padua, Italy; 4 National Council for Scientific and Technological Development (CNPq), Brasilia 71605-001, Brazil

**Keywords:** GxE, genotype by environment, reaction norm model, environmental sensitivity, robustness, Nellore, cattle, spline

## Abstract

**Simple Summary:**

In animal breeding, the different climatic conditions and production systems in beef cattle can result in the genotype by environment interaction. These interactions also need to be considered in genetic evaluations. In this study, five reaction norm models were tested on eleven traits related to growth, reproduction, and visual score measured in Nellore cattle breeding programs to check changes in the estimated genetic values of sires according to the selection environment. Using the best fitted statistical model, the presence of genotype by environment interaction was observed for some traits such as age at first calving, scrotal circumference, weaning to yearling weight gain, and yearling weight. For these traits, it is possible to select the best sires to increase productivity and reduce environmental sensitivity. Overall, the reaction norms trajectories for these traits seem to be affected by a non-linear component, and selecting robust animals for these traits is an alternative to increase production and reduce environmental sensitivity.

**Abstract:**

The assessment of the presence of genotype by environment interaction (GxE) in beef cattle is very important in tropical countries with diverse climatic conditions and production systems. The present study aimed to assess the presence of GxE by using different reaction norm models for eleven traits related to growth, reproduction, and visual score in Nellore cattle. We studied five reaction norm models (RNM), fitting a linear model considering homoscedastic residual variance (RNM_homo), and four models considering heteroskedasticity, being linear (RNM_hete), quadratic (RNM_quad), linear spline (RNM_l-l), and quadratic spline (RNM_q-q). There was the presence of GxE for age at first calving (AFC), scrotal circumference (SC), weaning to yearling weight gain (WYG), and yearling weight (YW). The best models were RNM_l-l for YW and RNM_q-q for AFC, SC, and WYG. The heritability estimates for RNM_l-l ranged from 0.07 to 0.20, 0.42 to 0.61, 0.24 to 0.42, and 0.47 to 0.63 for AFC, SC, WYG, and YW, respectively. The heteroskedasticity in reaction norm models improves the assessment of the presence of GxE for YW, WYG, AFC, and SC. Additionally, the trajectories of reaction norms for these traits seem to be affected by a non-linear component, and selecting robust animals for these traits is an alternative to increase production and reduce environmental sensitivity.

## 1. Introduction

The main concern of modern breeding programs in the tropics is the selection and mating of animals aiming to obtain progeny able to produce more efficiently under different production systems. Improvements in animal performance are directly related to genetic aspects and environmental conditions in which the animals are raised. Under tropical production systems, Nellore cattle are raised mainly on pasture under a wide range of climatic regions with seasonal variations in forage production and quality with differences in supplementation strategy [1] and temperature variation [2]. Such heterogeneous environmental factors have been recognized to directly affect animal performance and can provide a specific response from animals to changes in production systems [3,4,5].

The effect of genotype by environment interaction (GxE) has received special attention from several studies regarding its effect on traits related to growth, reproduction, and health [6,7,8,9,10]. These authors have pointed out the re-ranking of animals as the main interaction effect of GxE in tropical production systems for traits with economic importance in breeding programs. In Nellore cattle, dissecting the GxE interaction is commonly assessed by reaction norm models [9,11,12]. The reaction norm model allows fitting the phenotypic information as a function of a continuous environmental gradient by including covariates, regulated by environmental and genetic factors, which are shared with the target trait [13]. The reaction norm approach traditionally regresses the phenotypic value on the environmental gradients, assuming linear trajectories partitioning the estimated breeding value of animals into an independent component (intercept) and an environmental gradient dependent component (slope) associated with environmental sensitivity [14,15,16,17,18]. However, there is evidence that environmental sensitivity exhibits a non-linear trajectory across the environmental gradient. The GxE interaction evaluation using reaction norm models that contemplate the linear or non-linear trajectory is of utmost importance in finding the best model that fits the data based on environmental gradients to provide a more accurate genetic evaluation prediction [9]. 

The knowledge of the effects caused by GxE for traits of growth, reproduction, and visual scores, in addition, to the identification of linearity or not of the reaction norms trajectories, can be relevant to adjust strategies in breeding programs, considering the differences between environmental conditions of production systems. Therefore, more robust animals, i.e., slopes closer to zero and intercepts with high estimated breeding value (EBV) can be selected for herds with variation in the production levels [19]. Hence, the objective was to assess the presence of GxE by using different reaction norm models, contemplating linear and non-linear reaction norm trajectories, for growth, reproduction, and visual score traits evaluated in Nellore cattle taking into account goodness of fitting measures.

## 2. Material and Methods

### 2.1. Dataset Description

Approximately one million phenotypic records, collected from 1984 to 2016, from animals raised in different commercial farms located in four regions of Brazil (Southeast, Midwest, North, and Northeast) and Paraguay, were used in the present study. This data comprises a subset of the Alliance Nellore dataset (www.gensys.com.br (accessed on 24 September 2022)). The traits considered were birth to weaning weight gain (BWG), conformation (WC), finishing precocity (WP), muscling (WM) at weaning, yearling weight (YW), weaning to yearling weight gain (WYG), conformation (YC), finishing precocity (YP), muscling (YM) at yearling, scrotal circumference (SC), and age at first calving (AFC), which are part of the genetic evaluation of breeding programs.

Visual scores (WC, WP, WM, YC, YP, YM) were obtained from the evaluation of animals belonging to the same contemporary group (CG), i.e., animals that have had an equal opportunity to perform, by trained technicians. The technicians assess the scores of the animals within the CG applying relative scores from 1 to 5 for each trait (conformation, finishing precocity and muscling), where 1 means that the animal has low expression of the trait and 5 means the animal has maximum expression of the trait. Initially, the technicians determine the extremes 1 and 5 and the average animal (3) within each CG. Next, each animal is evaluated individually and scores are assigned. 

When measuring the conformation, the technician observes the meat production capacity in the carcass, imagining the slaughter at the time of evaluation, being observed mainly through the animal length, depth, and thoracic arch. Precocity is observed as the degree of finishing of the carcass, in which animals with good finishing precocity are those with good chest opening, good rib depth, heavy groin, and are at the beginning of subcutaneous fat deposition, especially at the base of the tail, combined with good body development. In muscularity, the development of muscular mass is evaluated as a whole.

Summary statistics of the dataset used for each trait after data editing are shown in Table 1. The data editing (removing the missing data and outliers) was carried out using R software [20].

The CG’s composition for each trait is described in Table 2. Only data from CGs with at least 20 animals were considered for the analyses. Birth year refers to the agricultural year (from July to June) in which the animal was born. The birth season was separated into two seasons, dry (autumn and winter), in which there is low rainfall, and rainy season (spring and summer), in which there is greater precipitation and, consequently, better pasture conditions. Besides considering CG as fixed effect, the model for weaning traits included the covariate age of animal at recording (as linear effect) and age of dam at calving (as linear and quadratic effects). For SC, the model included the covariate age of animal at recording as linear effect and for YW, YC, YP, and YM as linear and quadratic effects. For WYG, the model included the covariate period in days between weaning and yearling as linear and quadratic effects.

### 2.2. Environment Descriptor

When studying the GxE through reaction norm models, it is necessary to consider a continuous environmental descriptor in order to verify the animal response in each gradient. One way to define the environment descriptor is to use CG solutions previously estimated, once CG estimates reflect the environmental condition in which the contemporaries were raised [21]. Therefore, the environmental gradients (EGs) which were used to describe the level of production were based on CG solutions (obtained from model 1 or 2 described below) from birth to weaning weight gain for the weaning traits, on CG solutions from weaning to yearling weight gain for the visual score at yearling traits and WYG, and on CG solutions from yearling weight for YW, AFC, and SC. 

To estimate EGs, animal model 1 was used for weaning traits and animal model 2 for yearling traits.

(1) $$y_{w}=X_{w}b_{w}+Z_{w}a_{w}+M_{w}m_{w}+W_{w}c_{w}+e_{w}\;$$

(2) $$y_{y}=X_{y}b_{y}+Z_{y}a_{y}+e_{y}$$

where: $y_{w}$ and $y_{y}$ are the vectors of observations for BWG and WYG (or YW), respectively; $b_{w}$ and $b_{y}$ are the vector of fixed effects (contemporary group and covariates) at weaning and at yearling, respectively; $a_{w}$ and $a_{y}$ are the vectors of genetic additive effects; $m_{w}$ is a vector of random maternal genetic effects; $c_{w}$ is a vector of random maternal permanent environmental effects, $e_{w}$ and $e_{y}$ are the vectors of random residual effects; and $X_{w}$**,** $X_{y}$**,** $Z_{w}$**,** $Z_{y}$**,** $M_{w}$ and $W_{w}$ are incidence matrices related to $b_{w}$**,** $b_{y}$**,** $a_{w}$**,** $a_{y}$**,** $m_{w}$ and $c_{w}$, respectively.

The following assumptions were made for animal model 1:

(3) $$\left[a,m\right]\;\sim \;N\;(0,A⊗\left[\begin{array}{c} \begin{array}{cc} \sigma_{a}^{2\;} & \sigma_{am}\\ \sigma_{am} & \sigma_{m}^{2} \end{array} \end{array}\right])\;;\;c\;\sim \;N\left(0,I\sigma_{c}^{2}\right);\;e\;\sim \;N\left(0,\;I\sigma_{e}^{2}\right)$$

where: ***A*** is the numerator relationship matrix between animals; ***I*** is the identity matrix;$\;\sigma_{a}^{2}$ is the additive genetic variance, $\sigma_{m}^{2}$ is the maternal genetic variance; $\sigma_{c}^{2}$ is the maternal permanent environmental variance, $\sigma_{e}^{2}$ is the residual variance and $A\sigma_{am}$ is the genetic covariance between the additive genetic effect and the maternal genetic effect. For animal model 2, the following assumptions were made: $a\;\sim \;N(0,A\sigma_{a}^{2}$); $e\;\sim \;N(0,\;I\sigma_{e}^{2}$). The parameters of interest were estimated using the restricted maximum likelihood method implemented in AIREMLF90 program [22]. 

The CG solutions were standardized for a mean 0 and variance 1. Only records belonging to CG with standardized EG solutions between −3 and 3 sd were kept, in which after filtering, the minimum, average, and maximum standardized EG values for BWG and YW were equal to −3.0, 0.0, and 3.0 sd, and for WYG was equal to −2.77, 0.0, and 3.0 sd, corresponding to the respective effects of the CG solutions equal to 98.00, 152.75, and 207.36 kg for BWG, 168.52, 293.01 and 421.32 kg for YW, and 23.90, 104.27, and 191.11 kg for WYG. For AFC and SC, the minimum, average, and maximum standardized EG values for yearling weight were equal to −3.0, 0.0, and 3.0 sd and −2.5, 0.0, and 3.0 sd, respectively, corresponding to CG solutions equal to 195.71, 274.35, and 363.82 kg for AFC and 191.35, 308.03, and 426.70 kg for SC.

### 2.3. Reaction Norm Models (RNM)

A total of five reaction norm models were tested for the traits evaluated, as in [9]. 

The first model (RNM_homo) assumes homogeneity of residual variance for all EGs, as below:

(4) $$y_{ij}=X_{j}\beta +\emptyset {\hat{EG}}_{i}+b_{0}{}_{j}+b_{1}{}_{j}{\hat{EG}}_{i}+m_{j}+e_{ij}$$

where: $y_{ij}$ is the phenotypic data of animal *j* in given environment *i*, β is the vector of fixed effects and covariates; $X_{j}$ is the incidence matrix related to β; ∅ is the overall linear fixed regression coefficient of $y_{ij}$ in ${\hat{EG}}_{i}$; ${\hat{EG}}_{i}$ is the standardized EG solution of environment *i*, estimated previously; $b_{0}{}_{j}$ is the random direct additive genetic effect or the intercept of animal *j* for an average EG, $b_{1}{}_{j}$ is the coefficient of random regression or slope of the animal *j* in the environment represented by ${\hat{EG}}_{i}$; $m_{j}$ is the maternal genetic effect (considered only for the weaning traits), and $e_{ij}$ is residual effects. An attempt was made to also consider the maternal permanent environmental effect in the RNM for weaning traits, but the analyses did not converge. However, it is important to mention that the additive genetic and residual variances were little influenced by using a reduced model, without modeling the maternal permanent environmental effect (Table S1). The following assumptions were assumed for RNM_homo:

(5) $$\left[b_{0}{}_{j},b_{1}{}_{j}\right]\;\sim \;N(0,\;A⊗\left[\begin{array}{cc} \sigma_{b_{0}}^{2} & \sigma_{b_{0}b_{1}}\\ \sigma_{b_{0}b_{1}} & \sigma_{b_{1}}^{2} \end{array}\right]),\;m_{j}\;\sim N\left(0,A\sigma_{m}^{2}\right)\;\mathrm{and}\;e_{ij}\;\sim \;N\left(0,I\sigma_{e}^{2}\right),$$

where: ***A*** is the relationship matrix based on pedigree information (⊗ is the Kronecker product); $\sigma_{b_{0}}^{2}$, $\sigma_{b_{1}}^{2}$ and $\sigma_{b_{0}b_{1}}$ are the variances of the intercept, the slope and their covariance, respectively; $\sigma_{m}^{2}$ is the variance of the maternal genetic effect for weaning traits; ***I*** is an identity matrix and $\sigma_{e}^{2}$ is the residual variance.

The second model (RNM_hete) differed in relation to RNM_homo by assuming that the residual variance is heterogeneous across EGs, using a linear regression on ${\hat{EG}}_{i}$, with the intercept and slope coefficients being modeled using the log–residual function [23].

The third model (RNM_quad) also assumed heteroscedasticity, and considered a polynomial quadratic regression to model the fixed curve and the reaction norm of the random effects (additive genetic and residual) instead of linear regression, according to the equation:

(6) $$y_{ij}=X_{j}\beta +\emptyset_{1}{\hat{EG}}_{i}+\emptyset_{2}{\hat{EG}}_{i\;}^{\;2}+b_{0}{}_{j}+b_{1}{}_{j}{\hat{EG}}_{i}+b_{2}{}_{j}{\hat{EG}}_{i\;}^{\;2}+m_{j}+e_{ij},$$

where: $\emptyset_{2}$ is the quadratic fixed regression coefficient of $y_{ij}$ on ${\hat{EG}}_{i}$; ${\hat{EG}}_{i\;}^{\;2}$ is ${\hat{EG}}_{i}$ squared; $b_{2}{}_{j}$ is the quadratic effect of the additive genetic effect of the reaction norm of animal *j* on ${\hat{EG}}_{i}$ expressed as a deviation from $\emptyset_{2}$; the other terms were as described for RNM_homo.

The fourth model (RNM_l-l) also assumed heteroscedasticity, and used a linear–linear spline function to model the genetic merit of animals across the EG, using the same knot in the average EG (knot = 0) for all animals, according to the following equation:

(7) $$y_{ij}=X_{j}\beta +\emptyset_{1}{\hat{EG}}_{i}+\emptyset_{1}^{*}{\hat{EG}}_{i\;}^{\;*}+b_{0}{}_{j}+b_{1_{j}}{\hat{EG}}_{i}+b_{1}^{*}{}_{j}{\hat{EG}}_{i\;}^{\;*}+m_{j}+e_{ij},$$

where: $\emptyset_{1}^{*}$ is the difference of the regression coefficient between the first and the second segments of the linear–linear spline function of $y_{ij}$ on ${\hat{EG}}_{i\;}^{\;*}$, where ${\hat{EG}}_{i\;}^{\;*}$ is equal to zero if ${\hat{EG}}_{i}$ is less than or equal to zero, if ${\hat{EG}}_{i}$ is greater than zero, ${\hat{EG}}_{i\;}^{\;*}$ is equal to ${\hat{EG}}_{i}$; $b_{1}^{*}{}_{j}$ is the difference between the first and the second segments of the linear–linear spline function of the random additive genetic effect of animal *j* on ${\hat{EG}}_{i\;}^{\;*}$ expressed as a deviation from $\emptyset_{1}^{*}$; the other terms were described previously.

The fifth model (RNM_q-q) is similar to RNM_l-l, but considering a quadratic–quadratic spline function, according to the equation:

(8) $$y_{ij}=X_{j}\beta +\emptyset_{1}{\hat{EG}}_{i}+\emptyset_{2}{\hat{EG}}_{i\;}^{\;2}+\emptyset_{2}^{*}{\hat{EG}}_{i\;}^{\;2*}+b_{0}{}_{j}+b_{1_{j}}{\hat{EG}}_{i}+b_{2}{}_{j}{\hat{EG}}_{i\;}^{\;2}+\;b_{2}^{*}{}_{j}{\hat{EG}}_{i\;}^{\;2*}+m_{j}+e_{ij},$$

where: $\emptyset_{2}^{*}$ is the difference of the regression coefficient between the first and the second segments of the quadratic–quadratic spline function of $y_{ij}$ on $\emptyset_{2}^{*}{\hat{EG}}_{i\;}^{\;2*}$, where ${\hat{EG}}_{i\;}^{\;2*}$ is equal to zero if ${\hat{EG}}_{i}$ is less than or equal to zero, if ${\hat{EG}}_{i}$ is greater than zero, ${\hat{EG}}_{i\;}^{\;2*}$ is equal to ${\hat{EG}}_{i\;}^{\;2}$;$\;b_{2}^{*}{}_{j}$ is the difference between the first and the second segments of the quadratic–quadratic spline function of the random additive genetic effect of animal *j* on ${\hat{EG}}_{i\;}^{\;*}$ expressed as a deviation from $\emptyset_{2}^{*}$; the other terms were described previously.

The models RNM_quad, RNM_l-l, and RNM_q-q were also tested considering homogeneity of residual variance, but the heteroscedastic models outperformed them (results not shown). Because of this, was decided to keep only one homoscedastic model (RNM_homo).

In addition, as mentioned before, some analyses were performed considering different maternal random effects to understand the influence of including or omitting these effects in the animal model (Table S1).

### 2.4. Model Comparison

The models were compared based on the transformed values of AIC and Akaike weight (AICw), in which the interpretation of the values becomes straightforward, as it uses what would be the probability of choice of a model in relation to the other proposed models [24]. In addition, the plausibility of the biological interpretation of the results was verified, observing the trajectories of heritability estimates along the environmental gradient and the correlations between intercept and slopes. The heritability estimates were determined as the additive variance in each EG divided by the phenotypic variance (additive plus residual variances) in each EG.

All sires with reaction norms further inspected have at least fifty offspring and among these at least ten offspring in the low EG (−3 to −1) and ten in the high EG (1 to 3) or, for AFC, five offspring in the low and five in the high EG. 

Ten sires (top10) with the highest EBV in each environmental gradient (low, medium, and high) were selected to have their reaction norm described along the environmental gradient for YW, WYG, AFC, and SC. Additionally, forty sires with the highest EBV in the medium environmental gradient were selected. Of these, the ten most robust sires (top10 robust), i.e., with a slope closer to zero, were shown (Figures 5D–8D).

To assess the magnitude of GxE in different environments, Spearman correlations were calculated to compare the sire’s classification in the model with the best fit.

### 2.5. Environmental Sensitivity

A plasticity scale was assumed based on the absolute individual value of the slope ($b_{1_{j}}$) and standard deviation of the population slope ($\sigma_{b_{1}}$). The animals were classified as: robust $\left(\left|b_{1_{j}}\right|<\sigma_{b_{1}}\right)$, plastic $\left(\sigma_{b_{1}}<\left|b_{1_{j}}\right|<2\sigma_{b_{1}}\right)$, and extremely plastic $\left(\left|b_{1_{j}}\right|>2\sigma_{b_{1}}\right)$ [25].

## 3. Results

### 3.1. Reaction Norm Models

The slope/intercept ratios for slope and intercept variance estimates were low for all weaning traits (Table 3) and for yearling visual scores (Table S2). In addition, it is possible to note that for all weaning traits there is a low correlation between intercept and slope, especially for heteroscedastic models, with the exception of RNM_l-l and RNM_q-q, in which a high and negative correlation is observed between the first and second slope, suggesting a difference in the pattern of sensitivity from the first to the second segment (Table 3). According to model comparison criteria (AIC and AICw), for weaning traits (Table 3) and yearling visual scores (Table S2), the linear models (RNM_homo and RNM_hete) were the models that best fit the data.

For YW, WYG, SC, and AFC the slope/intercept ratio and the correlation between slope and intercept were higher than for the other traits (Table 4). For these traits, it can also be noted that there is a high and negative correlation between intercept and slope for RNM_l-l and RNM_q-q, which suggests a difference in the pattern of sensitivity between the first and second segments. The models that best fit the data according to AIC and AICw were RNM_l-l for YW and RNM_q-q for WYG, AFC, and SC (Table 4), which suggests that there is a non-linear component in the reaction norm for these traits. 

### 3.2. Heritability Estimates

**Figure 1:**
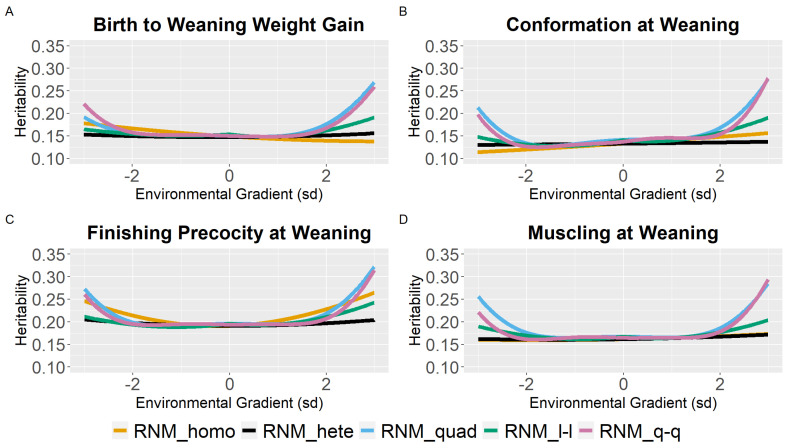
Heritability estimates (h^2^) for birth to weaning weight gain (**A**), conformation (**B**), finishing precocity (**C**), and muscling (**D**) at weaning traits in Nellore cattle according to the environmental gradient (standard deviation, sd), for different reaction norm models. RNM_homo: linear homoscedastic; RNM_hete: linear heteroscedastic; RNM_quad: quadratic heteroscedastic; RNM_l-l: spline linear–linear heteroscedastic; RNM_q-q: spline quadratic–quadratic heteroscedastic.

Weaning traits (BWG, WC, WP, and WM) showed similar heritability estimates (h^2^) for the different models between the intermediate gradients (−2 to 2 sd). However, a tendency for the overestimation of heritability estimates in the extreme gradients was observed for quadratic models. Furthermore, homoscedastic and linear heteroscedastic models presented similar heritability estimates for weaning traits (Figure 1; Table S3).

**Figure 2:**
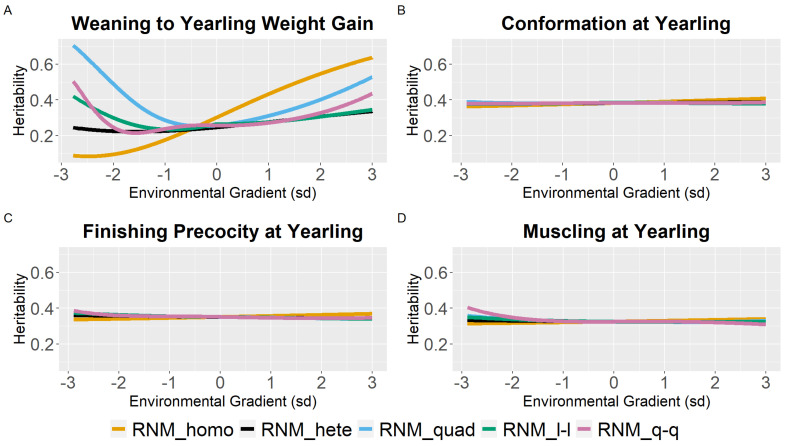
Heritability estimates (h^2^) for weaning to yearling weight gain (**A**), conformation (**B**), finishing precocity (**C**), and muscling (**D**) at yearling traits in Nellore cattle according to the environmental gradient, for different reaction norm models. RNM_homo: linear homoscedastic; RNM_hete: linear heteroscedastic; RNM_quad: quadratic heteroscedastic; RNM_l-l: spline linear–linear heteroscedastic; RNM_q-q: spline quadratic–quadratic heteroscedastic.

**Figure 3:**
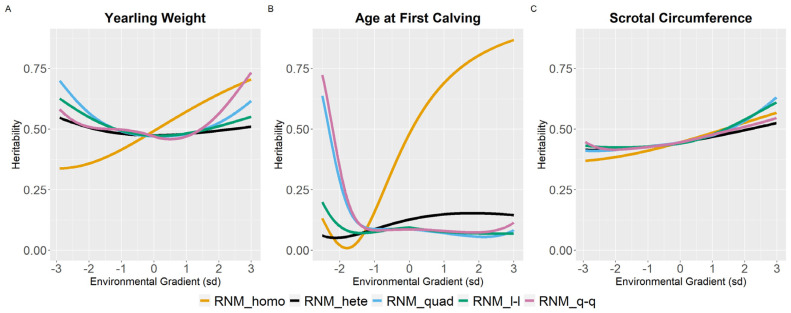
Heritability estimates (h^2^) for yearling weight (**A**), age at first calving (**B**) and scrotal circumference (**C**) in Nellore cattle according to the environmental gradient, for different reaction norm models. RNM_homo: linear homoscedastic; RNM_hete: linear heteroscedastic; RNM_quad: quadratic heteroscedastic; RNM_l-l: spline linear–linear heteroscedastic; RNM_q-q: spline quadratic–quadratic heteroscedastic.

For WYG, there are differences between models in the heritability estimates, mainly at the extreme gradients (Figure 2). For RNM_homo, the heritability estimates increased with the increase in EGs and tended to be underestimated in poor gradients and overestimated in better gradients when compared to models that assume heterogeneity of residual variance. Quadratic models (RNM_quad and RMN_q-q) resulted in an overestimation of heritability in extreme gradients for WYG. For YC, YP, and YM, similar heritability estimates were observed for all models (Figure 2), and the same occurred for SC (Figure 3). For YW, heritability estimates of different models followed a similar pattern as for WYG, where quadratic models overestimated heritability in extreme environments and RNM_homo underestimated heritability in poor gradients and overestimated it in better gradients (Figure 3). The greatest discrepancies in heritability estimates between models were observed for AFC (Figure 3). For this trait, RNM_homo overestimated heritability for better environments and quadratic models (RNM_quad and RMN_q-q) overestimated heritability for poor environments. 

### 3.3. Environmental Sensitivity 

**Figure 4:**
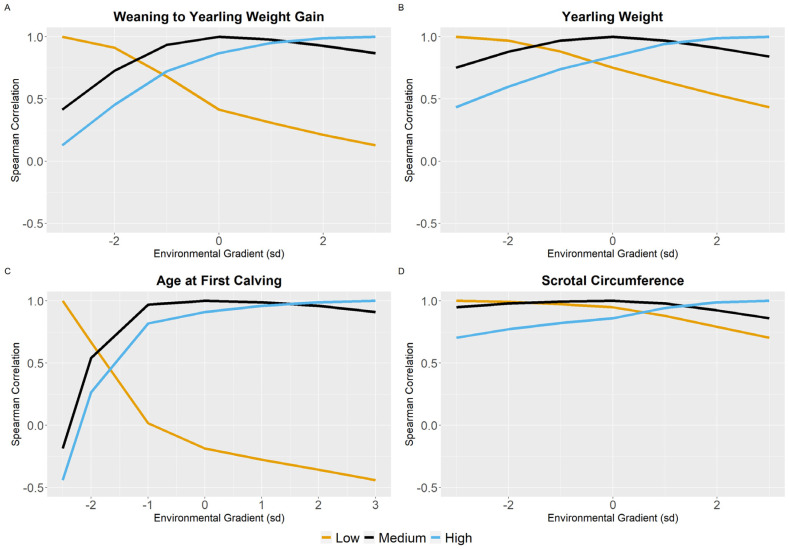
Estimates of Spearman correlation between sire’s EBVs for low, medium, and high gradients along the environmental gradient for weaning to yearling weight gain (**A**), yearling weight (**B**), age at first calving (**C**), and scrotal circumference (**D**).

Sires’ EBVs for medium and high EGs were highly correlated, even for traits with a more pronounced GxE effect (WYG, YW, AFC, SC), but low correlations were observed in contrasting EBVs for low with EBVs for medium and high EGs, especially for the traits WYG and AFC (Figure 4). High correlations among EBVs for different EGs were observed for the weaning and visual score traits (Figure S2). 

**Figure 5:**
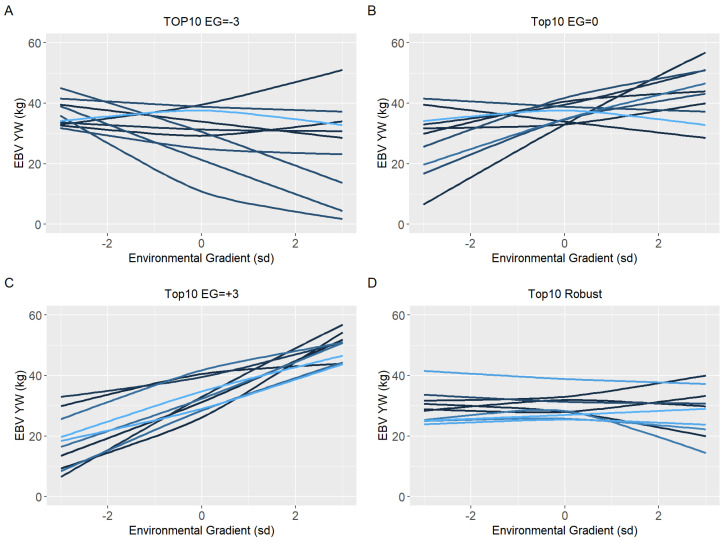
Estimated breeding values (EBV) reaction norms for yearling weight (YW) of Nellore cattle sires classified as top 10 for low (**A**), medium (**B**), and high environmental gradient (**C**), and top 10 robust (**D**), considering the reaction norm models (RNM) fitted with spline linear–linear heteroscedastic (RNM_l-l).

**Figure 6:**
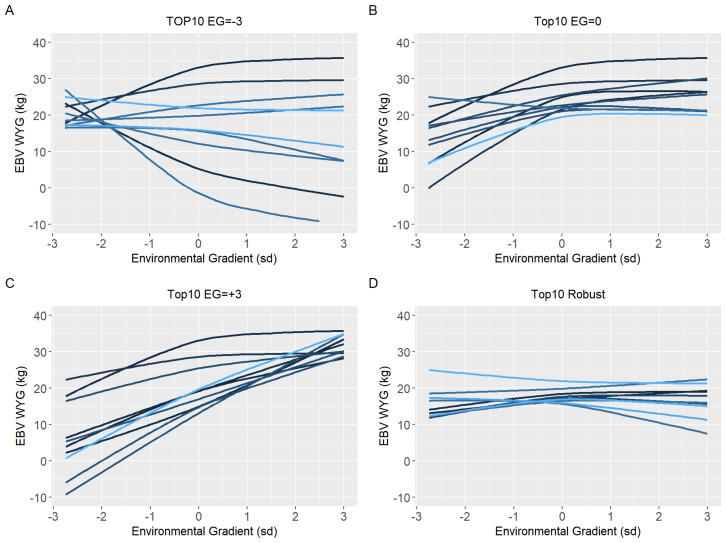
Estimated breeding values (EBV) reaction norms for weaning to yearling weight gain (WYG) of Nellore cattle sires classified as top 10 for low (**A**), medium (**B**), and high environmental gradient (**C**), and top 10 robust (**D**), considering the reaction norm models (RNM) fitted with spline linear–linear heteroscedastic (RNM_l-l).

**Figure 7:**
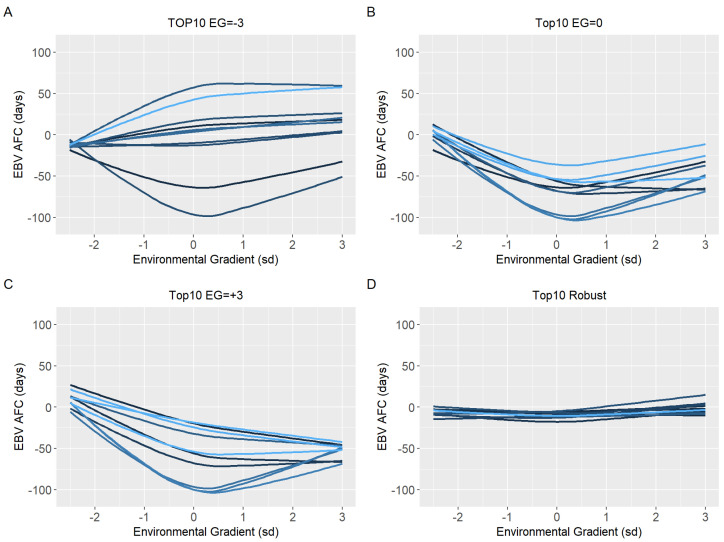
Estimated breeding values (EBV) reaction norms for age at first calving (AFC) of Nellore cattle sires classified as top 10 for low (**A**), medium (**B**), high EGs (**C**), and top 10 robust (**D**), considering the reaction norm models (RNM) fitted with spline linear–linear heteroscedastic (RNM_l-l).

**Figure 8:**
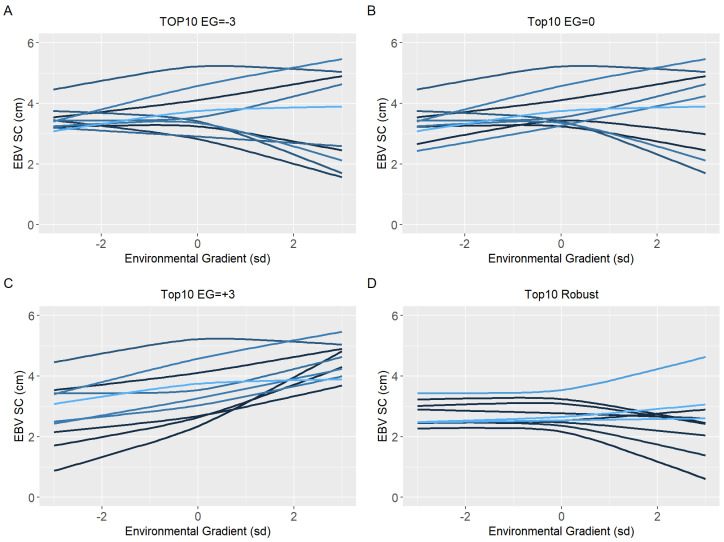
Estimated breeding values (EBV) reaction norms for scrotal circumference (SC) of Nellore cattle sires classified as top 10 for low (**A**), medium (**B**), and high environmental gradient (**C**), and top 10 robust (**D**) considering the reaction norm models (RNM) fitted with spline linear–linear heteroscedastic (RNM_l-l).

Because of the evidence of a non-linear component affecting the reaction norms and the overestimation of heritability for quadratic models (RNM_quad and RNM_q-q) in the extreme environments, it was decided to use results from RNM_l-l to further inspect the reaction norms of the top10 sires for WYG, YW, AFC, and SC (Figure 5, Figure 6, Figure 7 and Figure 8, respectively). It is possible to observe that there is a re-ranking of the top10 sires along the environmental gradients when selecting the top10 sires in the low (−3), medium (0), or high (+3) environmental gradients. The strategy of selecting sires tacking into account their EBVs for the average environment and also the variation of their EBVs between EGs (top10 robust sires) resulted in lower reranking, in comparison with selection for target environments (Figure 5, Figure 6, Figure 7 and Figure 8). As no evidence of GxE was found for weaning traits and yearling visual scores, the reaction norms for all sires in all models are presented as Supplementary Material (Figure S3–S13), as well top 10 corresponding figures for these traits (Figure S14–S20).

**Figure 9:**
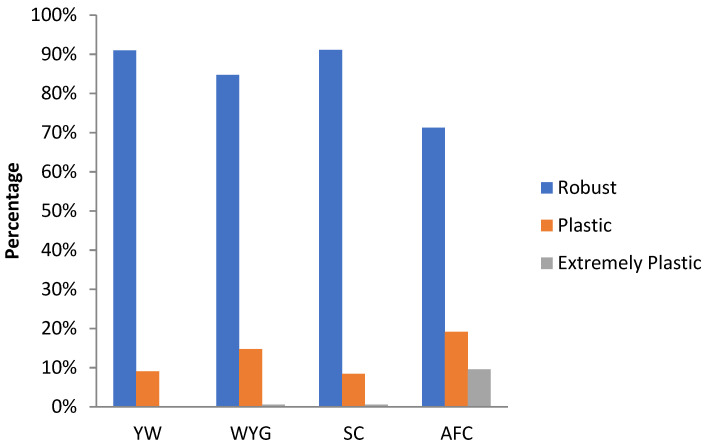
Percentage of animals with different genotypes (robust, plastic, and extremely plastic), for yearling weight (YW), weaning to yearling weight gain (WYG), scrotal circumference (SC) and age at first calving (AFC), considering the reaction norm models (RNM) fitted with spline linear–linear heteroscedastic (RNM_l-l).

The percentage of robust, plastic, and extremely plastic animals for YW, WYG, SC, and AFC are shown in Figure 9. For all traits, there was a predominance of robust animals according to the adopted criterium. AFC presented the highest proportion of plastic and extremely plastic animals among the studied traits.

## 4. Discussion

The identification of the presence of GxE can be performed by using multi-trait or reaction norms models [26]. In this study, five reaction norm models were evaluated for eleven traits (BWG, WC, WP, WM, YW, WYG, YC, YP, YM, SC, and AFC) in Nellore cattle raised under pasture conditions. The models were compared by the goodness of fitting considering the AIC and residual variance. Heritability estimates, reaction norms of EBVs, and environmental sensitivity were also analyzed.

### 4.1. Reaction Norm Models

For weaning traits and visual scores at yearling, the linear reaction norm models presented the best fit, suggesting that the animals present a single pattern of sensitivity along the environmental gradient. Ambrosini et al. [27], also observed that for weaning weight in Nellore cattle, the homoscedastic model outperformed the heteroscedastic model. However, as can be seen in the Table S1, the animal model surpassed the reaction norm models for WC, WP, and WM according to the AIC criteria, suggesting that there is no presence of GxE for these traits.

For YW, WYG, AFC, and SC, all heteroscedastic models outperformed the homoscedastic models, showing that heterogeneity of variances in different environments can be indicative of the presence of GxE [28]. Residual heterogeneity in the evaluation of GxE should be considered in the genetic evaluation of these traits in Nellore cattle to improve the partition of phenotypic variance in genetic and environmental components, enabling an increase in the response to selection [29]. For YW, AFC, and SC, Chiaia et al. [3] also found the superiority of the heteroscedastic model when compared with the homoscedastic.

For YW, the RNM_l-l model had the best fit and the RNM_q-q model had the best fit for WYG, SC, and AFC, suggesting that animal sensitivity will depend on the level of the environmental gradient, since some animals may be less sensitive to variation in more severe environmental gradients and more sensitive to variation in less challenging environmental gradients and vice versa [9].

According to Streit et al. [30], including residual variance heterogeneity in the model is important for obtaining unbiased genetic parameters. For YW, WYG, AFC, and SC, regardless of the model order in this study, heteroskedastic models were superior to homoscedastic models, mainly for AFC, where the genetic variance was highly overestimated as the environmental gradient improved in the homoscedastic model.

According to Meyer [31], choosing the best model is a compromise between what we might want to capture and the amount of information available in the data. Although quadratic models (RNM_quad e RNM_q-q) presented a better fit for the majority of the traits most affected by GxE (WYG, AFC, and SC), they tended to overestimate heritability for extreme environmental gradients. For this reason, RNM_l-l seems to be a good alternative model for a compromise between the goodness of fit and the plausibility of heritability estimates.

### 4.2. Heritability Estimates

Results obtained in this study illustrated that the heritability estimates varied along the environmental gradient (Figure 1, Figure 2 and Figure 3), due to variation in additive genetic and residual variance (for heteroscedastic models) as a function of the environmental gradient. Heritability estimates varying along EGs were also observed by Ambrosini et al. [32], Chiaia et al. [3], and Ribeiro et al. [33] for YW in Nellore cattle; by Chiaia et al. [3], Lemos et al. [34], and Mota et al. [12] for SC in Nellore cattle; by Chiaia et al. [3], Lemos et al. [34], and Araujo Neto et al. [4] for AFC in Nellore cattle; by Carvalheiro et al. [9] for adjusted weight gain from weaning to yearling in Nellore cattle; by Cardoso and Tempelman [35] for post-weaning weight gain in Hereford cattle; by Macneil, Cardoso, and Hay [36] for preweaning gain in Hereford cattle; and by Ambrosini et al. [27] for weaning weight in Nellore cattle.

In general, in low environmental conditions, there was an overestimation of the genetic variance for RNM_quad (Figure 1, Figure 2 and Figure 3). Trying to overcome this, an RNM_q-q was modeled as spline functions tend to be more robust against problems related to “end-of-range” estimates [37]. Nevertheless, the quadratic spline also overestimated heritability in extreme environments, which may be related to the low number of phenotypes observed in these environments (Figure S1), impairing the estimates at the extremes [37,38].

Heritability estimates for weaning traits and yearling visual scores, considering reaction norm models in the intermediate gradients (−2 to 2), were very similar to heritability estimates using the animal model (Figure 2 and Table S3), being another evidence of the lack of GxE for these traits. In addition, visual scores are attributed to CG, which may have impaired the estimation of GxE for those traits. Considering only the additive genetic effect in the reaction norm for weaning traits and yearling visual scores, heritability estimates were close to those found by Vargas et al. [39] using an animal model and a subset of the data used in the present study.

For weaning traits, it should be noted that the direct heritability estimates do not differ when using the complete model or the model with the exclusion of maternal permanent environment effect (Table S1). The maternal genetic variance absorbs maternal permanent environment variance, making this parameter overestimated, but the direct heritability estimate is not affected by the exclusion of this parameter.

Heritability estimates for SC had very similar trajectories for all models along the environmental gradient, indicating a higher opportunity for response to selection in better environments. This, however, would not necessarily result in a better-correlated response to sexual precocity, as there is evidence in the literature that SC is not a good indicator of sexual precocity in better environments [3].

In general, the environmental variation affects heritability estimates as a consequence of the GxE, and the understanding of the behavior of heritability estimates along the EGs is of great importance, because there may be a greater genetic gain for each environment.

### 4.3. Environmental Sensitivity

No difference was found between the environmental gradients when observing the genetic correlation between them (all traits >0.8), suggesting that there is no difference in the phenotypes of this population due to the environmental gradient. However, this can happen because it is a parameter of the entire population studied, in which many animals do not have enough information to estimate the EBV in several environmental gradients. However, when the Spearman correlation between a sire’s EBV is observed for YW, WYG, AFC, and SC (Figure 4), we can identify GxE in the studied gradients [40,41]. For visual score traits, this high correlation can be explained by the fact that the animals are evaluated within each CG, reducing the difference between each group.

Correlation estimates between the intercept and the slope(s) for the traits most affected by GxE were of medium to low magnitude, suggesting that animals are reclassified in different environments and that it is possible to carry out a combined selection in order to increase performance and reduce sensitivity [42,43]. However, even if the correlation between the intercept and the slope was high, the re-ranking of animals can still occur, as long as the EG interval is large enough and the slope variance is significantly different from zero because of the different trajectories of animal performance along the EGs are not parallel [44]. For YW (Table 3), WYG (Table 2), AFC (Table 3), and SC (Table 3), the correlations between intercept and slope were higher for the homoscedastic model and lower for the heteroscedastic models. Furthermore, it can be noted that the correlation between intercept and slope of the second segment for YW, WYG, AFC, and SC (Table 4) decreased compared with the correlation between intercept and slope of the first segment.

When a plastic animal is selected in the most favorable environment, its performance changes along the environmental gradient, as seen in Figure 5, Figure 6, Figure 7 and Figure 8, for YW, WYG, AFC, and SC. Consequently, an animal with lower EBV will be used in a medium or low environment, which would result in lower genetic gains. When trying to get around this, the selection of sires that will be parents in the next generation should consider a sire’s EBV for the environment in which its offspring will be raised.

Even though the re-ranking of sires among environmental gradients was expressive for some traits, most sires were classified as robust for YW, WYG, AFC, and SC (Figure 9), following the criteria adopted by Santana Jr et al. [25]. This highlights that observing the absolute value of the slope in relation to its standard deviation may not be an efficient criterion for identifying robust animals.

Mota et al. [5] suggested that selection is most effective when selecting the animal based on its EBV in the environment in which its progeny will be raised. Nevertheless, environmental conditions can vary substantially within a farm in different years, due to the seasonality of rainfall and, consequently, pasture conditions and differences in management. To overcome this, selection based on reaction norms becomes an important alternative for beef cattle breeding programs in the tropics, allowing joint selection for increased productivity and reduced sensitivity.

## 5. Conclusions

In Nellore cattle, it is not necessary to consider the presence of GxE in genetic evaluations for BWG and visual score traits (at weaning and yearling). On the other hand, for YW, WYG, SC, and AFC, accounting for GxE is important. Considering heteroskedasticity in reaction norm models improves the assessment of GxE for YW, WYG, AFC, and SC. In addition, the reaction norms for these traits seem to be affected by a non-linear component. The RMN_q-q model (best model according to the AIC criteria for WYG, AFC, and SC) overestimated the variances in the low environmental gradient. Therefore, among the tested models, RM_l-l seems the best option for genetic evaluation of YW, WYG, AFC, and SC in Nellore cattle. Furthermore, selecting robust animals for these traits is an alternative for increasing production and reducing environmental sensitivity.

## Figures and Tables

**Table 1:** Summary statistics of the dataset for each evaluated trait, considering the number of animals (N), number of males and females, minimum (Min), maximum (Max), mean (or median), standard deviation (sd), and the number of contemporary groups (CG).

Traits ^1^	N	Female	Male	Min	Mean	Max	sd	CG
BWG	553,381	276,994	276,387	70	157.2	278	32.95	11,657
WC	553,381	276,994	276,387	1	3 ^a^	5	-	11,657
WP	553,381	276,994	276,387	1	3 ^a^	5	-	11,657
WM	553,381	276,994	276,387	1	3 ^a^	5	-	11,657
YW	457,118	233,320	223,798	150	293	500	51.34	10,583
WYG	442,086	223,468	218,618	30	104.3	250	37.29	10,306
YC	529,673	270,252	259,421	1	3 ^a^	5	-	7246
YP	529,673	270,252	259,421	1	3 ^a^	5	-	7246
YM	529,673	270,252	259,421	1	3 ^a^	5	-	7246
SC	444,675	-	444,675	15	26.7	45	3.83	10,099
AFC	140,162	140,162	-	544	1012	1220	132	3897

^1^ BWG: birth to weaning weight gain; WC: conformation at weaning; WM; muscling at weaning; WP: precocity at weaning; YW: yearling weight; WYG: weaning to yearling weight gain; YC: conformation at yearling; YP: precocity at yearling; YM: muscling at yearling; SC: scrotal circumference; AFC: age at first calving. ^a^ Median.

**Table 2:** Variables included in the definition of contemporary groups.

Variable	Trait ^1^										
	BWG	WC	WP	WM	YW	WYG	YC	YP	YM	SC	AFC
Birth year	X	X	X	X	X	X	X	X	X	X	X
Birth season	X	X	X	X	X	X	X	X	X	X	X
Sex	X	X	X	X	X	X	X	X	X		
Farm at birth	X	X	X	X	X	X	X	X	X		
Farm at weaning	X	X	X	X	X	X				X	X
Weaning management group	X	X	X	X	X	X				X	X
Yearling management group					X	X	X	X	X	X	X
Farm at yearling					X	X	X	X	X	X	X

^1^ BWG: birth to weaning weight gain; WC: conformation at weaning; WP: precocity at weaning; WM: muscling at weaning; YW: yearling weight; WYG: weaning to yearling weight gain; YC: conformation at yearling; YP: precocity at yearling; YM: muscling at yearling; SC: scrotal circumference; AFC: age at first calving.

**Table 3:** Estimates of variance (diagonals), covariance (upper triangular; in italic), and correlation (lower triangular; in bold) among coefficients of reaction norm models (RNM) for the additive genetic effect of weaning traits in Nellore cattle, with respective residual variance estimates, Akaike information criterion (AIC), and AIC weight (AICw).

Trait ^1^	Model ^2^	Coefficient ^3^	b0	b1	b2	b3	σ^2^_m_	σ^2^_e_^4^	Np ^5^	AIC	AICw
BWG	RNM_homo	b0 (int)	55.67	*−1.51*				204.08	5	3,173,163.90	0.00
		b1 (slp)	**−0.29**	0.47							
		maternal					113.94				
	RNM_hete	b0 (int)	55.07	*−0.18*				5.32	6	3,173,157.20	0.00
		b1 (slp)	**−0.04**	0.35				−0.01			
		maternal					114.05				
	RNM_quad	b0 (int)	55.88	*−0.94*	*−0.83*			5.31	10	3173156.30	0.00
		b1 (slp)	**−0.11**	1.38	*0.45*			−0.02			
		b2 (qdr)	**−0.15**	**0.51**	0.57			0.01			
		maternal					114.06				
	RNM_l-l	b0 (int)	56.98	*0.84*	*−3.75*			5.30	10	3,173,160.00	0.00
		b1 (slp1)	**0.09**	1.61	*−1.58*			−0.04			
		b2 (slp2)	**−0.21**	**−0.53**	5.54			0.04			
		maternal					114.06				
	RNM_q-q	b0 (int)	55.77	*−1.95*	*−1.59*	*1.88*		5.31	15	3,173,130.20	1.00
		b1 (slp1)	**−0.14**	3.37	*1.48*	*−2.90*		−0.06			
		b2 (qdr1)	**−0.19**	**0.73**	1.23	*−1.69*		−0.02			
		b3 (qrd2)	**0.13**	**−0.83**	**−0.80**	3.66		0.06			
		maternal					114.00				
WC	RNM_homo	b0 (int)	0.1428	*0.0044*				0.7004	5	33,076.51	0.06
		b1 (slp)	**0.62**	0.0004							
		maternal					0.2300				
	RNM_hete	b0 (int)	0.1426	*0.0013*				−0.3559	6	33,070.84	0.94
		b1 (slp)	**0.30**	0.0001				0.0107			
		maternal					0.2301				
	RNM_quad	b0 (int)	0.1523	*0.0031*	*−0.0078*			−0.3649	10	33,257.81	0.00
		b1 (slp)	**0.09**	0.0077	*0.0007*			0.0055			
		b2 (qdr)	**−0.38**	**0.15**	0.0027			−0.0018			
		maternal					0.2304				
	RNM_l-l	b0 (int)	0.1510	*0.0121*	*−0.0177*			−0.3657	10	33,159.66	0.00
		b1 (slp1)	**0.33**	0.0091	*−0.0123*			0.0001			
		b2 (slp2)	**−0.28**	**−0.78**	0.0273			0.0117			
		maternal					0.2302				
	RNM_q-q	b0 (int)	0.1470	*0.0081*	*−0.0011*	*−0.0059*		−0.3640	15	33,210.89	0.00
		b1 (slp1)	**0.13**	0.0253	*0.0108*	*−0.0237*		0.0029			
		b2 (qdr1)	**−0.04**	**0.86**	0.0062	*−0.0113*		−0.0007			
		b3 (qrd2)	**−0.10**	**−0.93**	**−0.89**	0.0259		0.0022			
		maternal					0.2303				
WP	RNM_homo	b0 (int)	0.2349	*0.0026*				0.7900	5	104,255.53	0.55
		b1 (slp)	**0.33**	0.0003							
		maternal					0.2056				
	RNM_hete	b0 (int)	0.2349	*0.0014*				−0.2359	6	104,255.91	0.45
		b1 (slp)	**0.17**	0.0003				0.0049			
		maternal					0.2056				
	RNM_quad	b0 (int)	0.2426	*0.0006*	*−0.0076*			−0.2381	10	104,425.75	0.00
		b1 (slp)	**0.01**	0.0072	*0.0009*			0.0030			
		b2 (qdr)	**−0.29**	**0.19**	0.0029			−0.0066			
		maternal					0.2065				
	RNM_l-l	b0 (int)	0.2431	*0.0113*	*−0.0199*			−0.2358	10	104,334.27	0.00
		b1 (slp1)	**0.23**	0.0097	*−0.0148*			0.0072			
		b2 (slp2)	**−0.22**	**−0.82**	0.0337			−0.0118			
		maternal					0.2061				
	RNM_q-q	b0 (int)	0.2391	*−0.0006*	*−0.0044*	*0.0018*		−0.2380	15	104,375.02	0.00
		b1 (slp1)	**−0.01**	0.0199	*0.0092*	*−0.0192*		0.0023			
		b2 (qdr1)	**−0.12**	**0.84**	0.0060	*−0.0102*		−0.0063			
		b3 (qrd2)	**0.02**	**−0.92**	**−0.89**	0.0222		0.0037			
		maternal					0.2059				
WM	RNM_homo	b0 (int)	0.2078	*0.0017*				0.8269	5	128,693.45	0.73
		b1 (slp)	**0.12**	0.0009							
		maternal					0.2506				
	RNM_hete	b0 (int)	0.2078	*0.0013*				-0.1902	6	128,695.39	0.27
		b1 (slp)	**0.10**	0.0009				0.0011			
		maternal					0.2506				
	RNM_quad	b0 (int)	0.2160	*−0.0002*	*−0.0073*			−0.1937	10	128,882.36	0.00
		b1 (slp)	**−0.01**	0.0074	*0.0006*			0.0023			
		b2 (qdr)	**−0.29**	**0.13**	0.0029			−0.0051			
		maternal					0.2512				
	RNM_l-l	b0 (int)	0.2160	*0.0096*	*−0.0180*			−0.1945	10	128,781.97	0.00
		b1 (slp1)	**0.20**	0.0103	*−0.0150*			0.0015			
		b2 (slp2)	**−0.22**	**−0.83**	0.0320			0.0005			
		maternal					0.2508				
	RNM_q-q	b0 (int)	0.2119	*−0.0015*	*−0.0054*	*0.0030*		−0.1935	15	128,842.18	0.00
		b1 (slp1)	**−0.02**	0.0216	*0.0099*	*−0.0208*		0.0103			
		b2 (qdr1)	**−0.15**	**0.84**	0.0065	*−0.0110*		0.0035			
		b3 (qrd2)	**0.04**	**−0.92**	**−0.89**	0.0238		−0.0119			
		maternal					0.2511				

^1^ BWG: birth to weaning weight gain; WC: conformation at weaning; WP: finishing precocity at weaning; WM: muscling at weaning; ^2^ RNM_homo: linear homoscedastic; RNM_hete: linear heteroscedastic; RNM_quad: quadratic heteroscedastic; RNM_l-l: spline linear–linear heteroscedastic; RNM_q-q: spline quadratic–quadratic heteroscedastic; ^3^ b0–b3 coefficients of the RNM for the additive genetic random effect [int: intercept; slp: slope; qdr: quadratic; slp1(2): slope segment 1(2); qdr1(2): quadratic segment 1(2)] and maternal is the coefficients of the RNM for the maternal genetic random effect; ^4^ residual variance (RNM_homo) or residual coefficients associated with parameters of heteroscedastic RNM that were modeled using a log–residual function [23]; ^5^ number of estimated parameters.

**Table 4:** Estimates of variance (diagonals), covariance (upper triangular; in italic), and correlation (lower triangular; in bold) among coefficients of reaction norm models (RNM) for the additive genetic effect of yearling and reproductive traits in Nellore cattle, with respective residual variance estimates, Akaike information criterion (AIC), and AIC weight (AICw).

Traits ^1^	Model ^2^	Coefficient ^3^	b0	b1	b2	b3	σ^2^_e_^4^	Np ^5^	AIC	AICw
YW	RNM_homo	b0 (int)	350.81	*56.63*			361.68	4	4,164,832	0.00
		b1 (slp)	**0.69**	19.29						
	RNM_hete	b0 (int)	336.55	*23.99*			5.92	5	4,164,587	0.00
		b1 (slp)	**0.38**	12.14			0.14			
	RNM_quad	b0 (int)	336.43	*22.33*	*−1.68*		5.94	9	4,164,537	0.00
		b1 (slp)	**0.31**	15.23	*0.03*		0.15			
		b2 (qdr)	**−0.06**	**0.01**	1.98		−0.03			
	RNM_l-l	b0 (int)	343.00	*30.15*	*−16.51*		5.95	9	4,164,520	1.00
		b1 (slp1)	**0.34**	23.10	*−13.08*		0.19			
		b2 (slp2)	**−0.17**	**−0.52**	26.99		−0.09			
	RNM_q-q	b0 (int)	335.44	*8.72*	*−10.35*	*18.07*	5.94	14	4,164,531	0.00
		b1 (slp1)	**0.12**	17.05	*4.76*	*−6.06*	0.23			
		b2 (qdr1)	**−0.25**	**0.52**	4.97	*−7.02*	0.04			
		b3 (qrd2)	**0.27**	**−0.41**	**−0.87**	13.10	−0.13			
WYG	RNM_homo	b0 (int)	113.46	*35.97*			261.28	4	3,780,797	0.00
		b1 (slp)	**0.89**	14.49						
	RNM_hete	b0 (int)	92.02	*13.28*			5.64	5	3,780,155	0.00
		b1 (slp)	**0.59**	5.49			0.15			
	RNM_quad	b0 (int)	98.20	*15.19*	*−5.09*		5.65	9	3,779,938	0.00
		b1 (slp)	**0.47**	10.41	*−1.80*		0.17			
		b2 (qdr)	**−0.40**	**−0.44**	1.63		−0.03			
	RNM_l-l	b0 (int)	103.55	*26.12*	*−20.92*		5.66	9	3,779,976	0.00
		b1 (slp1)	**0.56**	20.68	*−15.95*		0.20			
		b2 (slp2)	**−0.46**	**−0.78**	20.06		−0.09			
	RNM_q-q	b0 (int)	97.78	*8.25*	*−7.79*	*6.07*	5.64	14	3,779,882	1.00
		b1 (slp1)	**0.21**	15.09	*8.03*	*−9.56*	0.22			
		b2 (qdr1)	**−0.27**	**0.72**	8.31	*−10.29*	0.03			
		b3 (qrd2)	**0.17**	**−0.68**	**−0.98**	13.24	−0.09			
AFC	RNM_homo	b0 (int)	3391.50	*1873.80*			3668.90	4	1,597,302	0.00
		b1 (slp)	**1.00**	1045.10						
	RNM_hete	b0 (int)	828.06	*309.89*			8.65	5	1,593,825	0.00
		b1 (slp)	**0.93**	133.86			0.46			
	RNM_quad	b0 (int)	651.88	*150.00*	*−107.08*		8.84	9	1,592,165	0.00
		b1 (slp)	**0.60**	94.51	*−31.44*		0.56			
		b2 (qdr)	**−0.82**	**−0.64**	25.86		−0.15			
	RNM_l-l	b0 (int)	746.31	*331.13*	*−354.60*		8.88	9	1,592,410	0.00
		b1 (slp1)	**0.89**	187.18	*−171.16*		0.77			
		b2 (slp2)	**−0.89**	**−0.86**	210.83		−0.52			
	RNM_q-q	b0 (int)	631.40	*178.12*	*−61.62*	*−46.08*	8.82	14	1,592,007	1.00
		b1 (slp1)	**0.62**	131.68	*26.11*	*−59.51*	0.54			
		b2 (qdr1)	**−0.30**	**0.28**	67.02	*−64.41*	−0.17			
		b3 (qrd2)	**−0.20**	**−0.56**	**−0.85**	86.62	0.02			
SC	RNM_homo	b0 (int)	3.03	*0.23*			3.78	4	2,059,717.9	0.00
		b1 (slp)	**0.53**	0.06						
	RNM_hete	b0 (int)	3.02	*0.17*			1.33	5	2,059,691.4	0.00
		b1 (slp)	**0.43**	0.05			0.03			
	RNM_quad	b0 (int)	3.06	*0.15*	*−0.04*		1.34	9	2,059,612.4	0.00
		b1 (slp)	**0.26**	0.11	*0.02*		0.02			
		b2 (qdr)	**−0.28**	**0.56**	0.01		−0.02			
	RNM_l-l	b0 (int)	3.07	*0.20*	*−0.13*		1.36	9	2,059,618.7	0.00
		b1 (slp1)	**0.46**	0.06	*−0.01*		0.07			
		b2 (slp2)	**−0.18**	**−0.07**	0.18		−0.10			
	RNM_q-q	b0 (int)	3.05	*0.14*	*−0.06*	*0.04*	1.34	14	2,059,596.1	1.00
		b1 (slp1)	**0.18**	0.20	*0.06*	*−0.08*	−0.02			
		b2 (qdr1)	**−0.20**	**0.72**	0.03	*−0.04*	−0.04			
		b3 (qrd2)	**0.09**	**−0.78**	**−0.90**	0.06	0.05			

^1^ YW: yearling weight; WYG: weaning to yearling weight gain; AFC: age at first calving; SC: scrotal circumference; ^2^ RNM_homo: linear homoscedastic; RNM_hete: linear heteroscedastic; RNM_quad: quadratic heteroscedastic; RNM_l-l: spline linear–linear heteroscedastic; RNM_q-q: spline quadratic–quadratic heteroscedastic; ^3^ b0–b3 coefficients of the RNM for the additive genetic random effect [int: intercept; slp: slope; qdr: quadratic; slp1(2): slope segment 1(2); qdr1(2): quadratic segment 1(2)]; ^4^ residual variance (RNM_homo) or residual coefficients associated with parameters of heteroscedastic RNM that were modeled using a log–residual function [23].; ^5^ number of estimated parameters.

## Data Availability

The data sets employed in this study are the property of the Genetic Breeding Programs participating in the Aliança Nellore and cannot be shared. All the data supporting the results of this study are included in the article and in the Supplementary Files.

## Supplementary Materials

The following are available online at https://www.mdpi.com/article/10.3390/ani12192613/s1, Table S1: variance estimates and heritability for weaning and yearling traits in Nellore cattle according to the animal model used; Table S2: estimates of variance (diagonals), covariance (upper triangular; in italic), and correlation (lower triangular; in bold) among coefficients of reaction norm models (RNM) for the additive genetic effect of yearling visual score traits in Nellore cattle, with respective residual variance estimates, Akaike information criterion (AIC) and AIC weight (AICw); Table S3: average (minimum and maximum) heritability estimates for weaning, yearling, and reproductive traits in Nellore cattle for different reaction norm models; Figure S1: number of phenotypes by environmental gradients for birth to weaning weight gain (A), weaning to yearling weight gain (B), weaning to yearling weight gain used for yearling visual scores (C), yearling weight (D), yearling weight used for age at first calving (E), and yearling weight used for scrotal circumference (F); Figure S2: estimates of Spearman correlation between sire’s EBVs for low, medium, and high gradients along the environmental gradient for birth to weaning weight gain (A), conformation at weaning (B), finishing precocity at weaning (C), muscling ate weaning (D), conformation at yearling (E), finishing precocity at yearling (F), and muscling at yearling (G); Figure S3: estimated breeding values (EBV) as function of environmental gradient of sires with at least 50 progenies (*n* = 355) for yearling weight (YW) in Nellore cattle by using different reaction norm models. RNM_homo (A): linear homoscedastic; RNM_hete (B): linear heteroscedastic; RNM_quad (C): quadratic heteroscedastic; RNM_l-l (D): spline linear–linear heteroscedastic; RNM_q-q (E): spline quadratic–quadratic heteroscedastic. EBV reaction norms (F) of three selected sires (differentiated by color) for models RNM_homo (solid line), RNM_hete (two dash line), RNM_quad (dotted curve), RNM_l-l (long dash curve), and RNM_q-q (dot dash curve); Figure S4: estimated breeding values (EBV) as function of environmental gradient sires with at least 50 progenies (*n* = 380) for weaning to yearling weight gain (WYG) in Nellore cattle by using different reaction norm models. RNM_homo (A): linear homoscedastic; RNM_hete (B): linear heteroscedastic; RNM_quad (C): quadratic heteroscedastic; RNM_l-l (D): spline linear–linear heteroscedastic; RNM_q-q (E): spline quadratic–quadratic heteroscedastic. EBV reaction norms (F) of three selected sires (differentiated by color) for models RNM_homo (solid line), RNM_hete (two dash line), RNM_quad (dotted curve), RNM_l-l (long dash curve), and RNM_q-q (dot dash curve); Figure S5: estimated breeding values (EBV) as function of environmental gradient of sires with at least 50 progenies (*n* = 94) for age at first calving (AFC) in Nellore cattle by using different reaction norm models. RNM_homo (A): linear homoscedastic; RNM_hete (B): linear heteroscedastic; RNM_quad (C): quadratic heteroscedastic; RNM_l-l (D): spline linear–linear heteroscedastic; RNM_q-q (E): spline quadratic–quadratic heteroscedastic. EBV reaction norms (F) of three selected sires (differentiated by color) for models RNM_homo (solid line), RNM_hete (two dash line), RNM_quad (dotted curve), RNM_l-l (long dash curve), and RNM_q-q (dot dash curve); Figure S6: estimated breeding values (EBV) as function of environmental gradient of sires with at least 50 progenies (*n* = 191) for scrotal circumference (SC) in Nellore cattle by using different reaction norm models. RNM_homo (A): linear homoscedastic; RNM_hete (B): linear heteroscedastic; RNM_quad (C): quadratic heteroscedastic; RNM_l-l (D): spline linear–linear heteroscedastic; RNM_q-q (E): spline quadratic–quadratic heteroscedastic. EBV reaction norms (F) of three selected sires (differentiated by color) for models RNM_homo (solid line), RNM_hete (two dash line), RNM_quad (dotted curve), RNM_l-l (long dash curve), and RNM_q-q (dot dash curve); Figure S7: estimated breeding values (EBV) as function of environmental gradient of sires with at least 50 progenies (*n* = 724) for birth to weaning weight gain (BWG) in Nellore cattle by using different reaction norm models. RNM_homo (A): linear homoscedastic; RNM_hete (B): linear heteroscedastic; RNM_quad (C): quadratic heteroscedastic; RNM_l-l (D): spline linear–linear heteroscedastic; RNM_q-q (E): spline quadratic–quadratic heteroscedastic. EBV reaction norms (F) of three selected sires (differentiated by color) for models RNM_homo (solid line), RNM_hete (two dash line), RNM_quad (dotted curve), RNM_l-l (long dash curve), and RNM_q-q (dot dash curve); Figure S8: estimated breeding values (EBV) as function of environmental gradient of sires with at least 50 progenies (*n* = 724) for conformation at weaning (WC) in Nellore cattle by using different reaction norm models. RNM_homo (A): linear homoscedastic; RNM_hete (B): linear heteroscedastic; RNM_quad (C): quadratic heteroscedastic; RNM_l-l (D): spline linear–linear heteroscedastic; RNM_q-q (E): spline quadratic–quadratic heteroscedastic. EBV reaction norms (F) of three selected sires (differentiated by color) for models RNM_homo (solid line), RNM_hete (two dash line), RNM_quad (dotted curve), RNM_l-l (long dash curve), and RNM_q-q (dot dash curve); Figure S9: estimated breeding values (EBV) as function of environmental gradient of sires with at least 50 progenies (*n* = 724) for precocity at weaning (WP) in Nellore cattle by using different reaction norm models. RNM_homo (A): linear homoscedastic; RNM_hete (B): linear heteroscedastic; RNM_quad (C): quadratic heteroscedastic; RNM_l-l (D): spline linear–linear heteroscedastic; RNM_q-q (E): spline quadratic–quadratic heteroscedastic. EBV reaction norms (F) of three selected sires (differentiated by color) for models RNM_homo (solid line), RNM_hete (two dash line), RNM_quad (dotted curve), RNM_l-l (long dash curve), and RNM_q-q (dot dash curve); Figure S10: estimated breeding values (EBV) as function of environmental gradient of sires with at least 50 progenies (*n* = 724) for muscling at weaning (WM) in Nellore cattle by using different reaction norm models. RNM_homo (A): linear homoscedastic; RNM_hete (B): linear heteroscedastic; RNM_quad (C): quadratic heteroscedastic; RNM_l-l (D): spline linear–linear heteroscedastic; RNM_q-q (E): spline quadratic–quadratic heteroscedastic. EBV reaction norms (F) of three selected sires (differentiated by color) for models RNM_homo (solid line), RNM_hete (two dash line), RNM_quad (dotted curve), RNM_l-l (long dash curve), and RNM_q-q (dot dash curve); Figure S11: estimated breeding values (EBV) as function of environmental gradient of sires with at least 50 progenies (*n* = 557) for conformation at yearling (YC) in Nellore cattle by using different reaction norm models. RNM_homo (A): linear homoscedastic; RNM_hete (B): linear heteroscedastic; RNM_quad (C): quadratic heteroscedastic; RNM_l-l (D): spline linear–linear heteroscedastic; RNM_q-q (E): spline quadratic–quadratic heteroscedastic. EBV reaction norms (F) of three selected sires (differentiated by color) for models RNM_homo (solid line), RNM_hete (two dash line), RNM_quad (dotted curve), RNM_l-l (long dash curve), and RNM_q-q (dot dash curve); Figure S12: estimated breeding values (EBV) as function of environmental gradient of sires with at least 50 progenies (*n* = 557) for precocity at yearling (YP) in Nellore cattle by using different reaction norm models. RNM_homo (A): linear homoscedastic; RNM_hete (B): linear heteroscedastic; RNM_quad (C): quadratic heteroscedastic; RNM_l-l (D): spline linear–linear heteroscedastic; RNM_q-q (E): spline quadratic–quadratic heteroscedastic. EBV reaction norms (F) of three selected sires (differentiated by color) for models RNM_homo (solid line), RNM_hete (two dash line), RNM_quad (dotted curve), RNM_l-l (long dash curve), and RNM_q-q (dot dash curve); Figure S13: estimated breeding values (EBV) as function of environmental gradient of sires with at least 50 progenies (*n* = 557) for muscling at yearling (YM) in Nellore cattle by using different reaction norm models. RNM_homo (A): linear homoscedastic; RNM_hete (B): linear heteroscedastic; RNM_quad (C): quadratic heteroscedastic; RNM_l-l (D): spline linear–linear heteroscedastic; RNM_q-q (E): spline quadratic–quadratic heteroscedastic. EBV reaction norms (F) of three selected sires (differentiated by color) for models RNM_homo (solid line), RNM_hete (two dash line), RNM_quad (dotted curve), RNM_l-l (long dash curve), and RNM_q-q (dot dash curve); Figure S14: estimated breeding values (EBV) reaction norms for birth to weaning weight gain (BWG) of Nellore cattle sires classified as top 10 for low (A), medium (B), and high environmental gradient (C), and top 10 robust (D) considering the reaction norm models (RNM) fitted with spline linear–linear heteroscedastic (RNM_l-l); Figure S15: estimated breeding values (EBV) reaction norms for conformation at weaning (WC) of Nellore cattle sires classified as top 10 for low (A), medium (B), and high environmental gradient (C), and top 10 robust (D) considering the reaction norm models (RNM) linear heteroscedastic (RNM_hete); Figure S16: estimated breeding values (EBV) reaction norms for precocity at weaning (WP) of Nellore cattle sires classified as top 10 for low (A), medium (B), high environmental gradient (C), and top 10 robust (D) considering the reaction norm models (RNM) linear homoscedastic (RNM_homo); Figure S17: estimated breeding values (EBV) reaction norms for muscling at weaning (WM) of Nellore cattle sires classified as top 10 for low (A), medium (B), high environmental gradient (C), and top 10 robust (D) considering the reaction norm models (RNM) linear homoscedastic (RNM_homo); Figure S18: estimated breeding values (EBV) reaction norms for conformation at yearling (YC) of Nellore cattle sires classified as top 10 for low (A), medium (B), high environmental gradient (C), and top 10 robust (D) considering the reaction norm models (RNM) linear heteroscedastic (RNM_hete); Figure S19: estimated breeding values (EBV) reaction norms for precocity at yearling (YP) of Nellore cattle sires classified as top 10 for low (A), medium (B), high environmental gradient (C), and top 10 robust (D) considering the reaction norm models (RNM) linear heteroscedastic (RNM_hete); Figure S20: estimated breeding values (EBV) reaction norms for muscling at yearling (YM) of Nellore cattle sires classified as top 10 for low (A), medium (B), high environmental gradient (C), and top 10 robust (D) considering the reaction norm models (RNM) linear heteroscedastic (RNM_hete).

## References

[1] Poppi D.P., Quigley S.P., da Silva T.A.C.C., McLennan S.R. (2018). Challenges of beef cattle production from tropical pastures. Rev. Bras. Zootec..

[2] Alvares C.A., Stape J.L., Sentelhas P.C., De Moraes Gonçalves J.L., Sparovek G. (2013). Köppen’s climate classification map for Brazil. Meteorol. Z..

[3] Chiaia H.L.J., De Lemos M.V.A., Venturini G.C., Aboujaoude C., Berton M.P., Feitosa F.B., Carvalheiro R., Albuquerque L.G., de Oliveira H.N., Baldi F. (2015). Genotype × environment interaction for age at first calving, scrotal circumference, and yearling weight in Nellore cattle using reaction norms in multitrait random regression models. J. Anim. Sci..

[4] de Araujo Neto F.R., Pegolo N.T., Aspilcueta-borquis R.R., Pessoa M.C., Bonifácio A., Lobo R.B., de Oliveira H.N. (2018). Study of the effect of genotype × environment interaction on age at first calving and production traits in Nellore cattle using multi-trait reaction norms and Bayesian inference. Anim. Sci. J..

[5] Mota L.F.M., Costa L.S., Garzón N.A.M., Passafaro T.L., Silva D.O., Abreu L.R.A., Verardo L.L., Bonafé C.M., Ventura H.T. (2019). Unraveling the effect of body structure score on phenotypic plasticity for body weight at di ff erent ages in Guzerat cattle. Livest. Sci..

[6] Ambrosini D.P., Henrique C., Malhado M., Filho R.M., Cardoso F.F., Luiz P., Carneiro S. (2016). Genotype × environment interactions in reproductive traits of Nellore cattle in northeastern Brazil. Trop. Anim. Health Prod..

[7] Mota R.R., Tempelman R.J., Lopes P.S., Aguilar I., Silva F.F., Cardoso F.F. (2016). Genotype by environment interaction for tick resistance of Hereford and Braford beef cattle using reaction norm models. Genet. Sel. Evol..

[8] Oliveira D.P., Lourenco D.A.L., Tsuruta S., Misztal I., Santos D.J.A., de Araújo Neto F.R., Aspilcueta-Borquis R.R., Baldi F., Carvalheiro R., de Camargo G.M.F. (2018). Reaction norm for yearling weight in beef cattle using single-step genomic evaluation1. J. Anim. Sci..

[9] Carvalheiro R., Costilla R., Neves H.H.R., Albuquerque L.G., Moore S., Hayes B.J. (2019). Unraveling genetic sensitivity of beef cattle to environmental variation under tropical conditions. Genet. Sel. Evol..

[10] Carvalho C.V.D., Costa R.B., de Camargo G.M.F., Bittencourt T.C.C. (2019). Genotype × Environment Interaction for reproductive traits in brazilian Nellore breed cattle. Rev. Bras. Saude e Prod. Anim..

[11] Silva T.D.L., Carneiro P.L.S., Ambrosini D.P., Lobo R.B., Martins Filho R., Malhado C.H.M. (2019). Genotype-environment interaction in the genetic variability analysis of reproductive traits in Nellore cattle. Livest. Sci..

[12] Mota L.F.M., Fernandes G.A., Herrera A.C., Scalez D.C.B., Espigolan R., Carvalheiro R., Baldi F., Albuquerque L.G. (2020). Genomic reaction norm models exploiting genotype × environment interaction on sexual precocity indicator traits in Nellore cattle. Anim. Genet..

[13] Schaeffer L.R. (2004). Application of random regression models in animal breeding. Livest. Prod. Sci..

[14] Rezende M.P.G., Malhado C.H.M., Biffani S., Carneiro P.L.S., Carrillo J.A., Bozzi R. (2020). Genotype-environment interaction for age at first calving in Limousine and Charolais cattle raised in Italy, employing reaction norm model. Livest. Sci..

[15] Toghiani S., Hay E., Fragomeni B., Rekaya R., Roberts A.J. (2020). Genotype by environment interaction in response to cold stress in a composite beef cattle breed. Animal.

[16] Freitas A.D.P., Santana Júnior M.L., Schenkel F.S., Mercadante M.E.Z., Cyrillo J.N.D.S.G., de Paz C.C.P. (2021). Different selection practices affect the environmental sensitivity of beef cattle. PLoS ONE.

[17] Negri R., Aguilar I., Feltes G.L., Cobuci J.A. (2021). Selection for Test-Day Milk Yield and Thermotolerance in Brazilian Holstein Cattle. Animals.

[18] Shi R., Brito L.F., Liu A., Luo H., Chen Z., Liu L., Guo G., Mulder H., Ducro B., van der Linden A. (2021). Genotype-by-environment interaction in Holstein heifer fertility traits using single-step genomic reaction norm models. BMC Genom..

[19] Strandberg E., Kolmodin R., Madsen P., Jensen J., Jorjani H. (2000). Genotype by Environment Interaction in Nordic Dairy Cattle Studied by Use of Reaction Norms. Interbull Bull..

[20] R Core Team (2022). A Language and Environment for Statistical Computing.

[21] Kolmodin R., Strandberg E., Madsen P., Jensen J., Jorjani H. (2002). Genotype by Environment Interaction in Nordic Dairy Cattle Studied Using Reaction Norms. Acta Agric. Scand..

[22] Misztal I., Tsuruta S., Strabel T., Auvray B., Druet T., Lee D.H. In Proceedings of the 7th World Congress on Genetics Applied to Livestock Production.

[23] Foulley J.L., Quaas R.L. (1995). Heterogeneous variances in Gaussian linear mixed models. Genet. Sel. Evol..

[24] Burnham K.P., Anderson D.R. (2004). Multimodel Inference Understanding AIC and BIC in Model Selection. Sociol. Methods Res..

[25] Sanata M.L., Bignardi A.B., Pereira R.J. (2016). Random regression models to account for the effect of genotype by environment interaction due to heat stress on the milk yield of Holstein cows under tropical conditions. J. Appl. Genet..

[26] Hayes B.J., Daetwyler H.D., Goddard M.E. (2016). Models for Genome × Environment interaction: Examples in livestock. Crop Sci..

[27] Ambrosini D.P., Malhado C.H.M., Braccini Neto J., Martins Filho R., Affonso P.R.A.D.M., Luiz P.S.C. (2014). Reaction norms of direct and maternal effects for weight at 205 days in Polled Nellore cattle in north-eastern Brazil. Arch. Tierz..

[28] Calus M.P.L., Groen A.F., De Jong G. (2002). Genotype × Environment Interaction for Protein Yield in Dutch Dairy Cattle as Quantified by Different Models. J. Dairy Sci..

[29] Cardoso L.L., Neto J.B., Cardoso F.F., Araújo J., Biassus I.D.O., Otávio J., Barcellos J. (2011). Hierarchical Bayesian models for genotype × environment estimates in post-weaning gain of Hereford bovine via reaction norms. Rev. Bras. Zootec..

[30] Streit M., Reinhardt F., Thaller G., Bennewitz J. (2012). Reaction norms and genotype-by-environment interaction in the German Holstein dairy cattle. J. Anim. Breed. Genet..

[31] Meyer K. (2005). Estimates of genetic covariance functions for growth of Angus cattle. J. Anim. Breed. Genet..

[32] Ambrosini D.P., Carneiro P.L.S., Bracicini Neto J., Martins Filho R., Amaral R.D.S., Cardoso F.F., Malhado C.H.M. (2014). Reaction norms models in the adjusted weight at 550 days of age for Polled Nellore cattle in Northeast Brazil. Rev. Bras. Zootec..

[33] Ribeiro S., Eler J.P., Pedrosa V.B., Rosa G.J.M., Ferraz J.B.S., Balieiro J.C.C. (2017). Genotype by environment interaction for yearling weight in Nellore cattle applying reaction norms models. Anim. Prod. Sci..

[34] Lemos M.V.A., Chiaia H.L.J., Berton M.P., Feitosa F.L.B., Aboujaoude C., Venturini G.C., Oliveira H.N., Albuquerque L.G., Baldi F. (2015). Reaction norms for the study of genotype- environment interaction for growth and indicator traits of sexual precocity in Nellore cattle. Genet. Mol. Res..

[35] Cardoso F.F., Tempelman R.J. (2012). Linear reaction norm models for genetic merit prediction of Angus cattle under genotype by environment interaction. J. Anim. Sci..

[36] Macneil M.D., Cardoso F.F., Hay E. (2017). Genotype by environment interaction effects in genetic evaluation of preweaning gain for Line 1 Hereford cattle from Miles City, Montana 1. J. Anim. Sci..

[37] Meyer K. (2005). Random regression analyses using B-splines to model growth of Australian Angus cattle. Genet. Sel. Evol..

[38] Misztal I. (2006). Properties of random regression models using linear splines. J. Anim. Breed. Genet..

[39] Vargas G., Schenkel F.S., Brito L.F. (2018). Unravelling Biological Biotypes for Growth, Visual Score and Reproductive Traits in Nellore Cattle via Principal Component Analysis. Livest. Sci..

[40] Sigurdsson A., Banos G., Philipsson J. (1996). Estimation of Genetic (Co)variance Components for International Evaluation of Dairy Bulls. Acta Agric. Scand. Sect. A—Anim. Sci..

[41] Hammami H., Rekik B., Soyeurt H., Bastin C., Bay E., Stoll J., Gengler N. (2009). Accessing genotype by environment interaction using within- and across-country test-day random regression sire models. J. Anim. Breed. Genet..

[42] Santana M.L., Eler J.P., Cardoso F.F., Albuquerque L.G., Ferraz J.B.S. (2013). Phenotypic plasticity of composite beef cattle performance using reaction norms model with unknown covariate. Animal.

[43] Su G., Madsen P., Lund M.S., Sorensen D., Korsgaard I.R., Jensen J. (2006). Bayesian analysis of the linear reaction norm model with unknown covariates Bayesian analysis of the linear reaction norm model with unknown covariates 1. J. Anim. Sci..

[44] Zhang Z., Kargo M., Liu A., Thomasen J.R., Pan Y., Su G. (2019). Genotype-by-environment interaction of fertility traits in Danish Holstein cattle using a single-step genomic reaction norm model. Heredity.

