# Supplementary material for: Heteroscedastic Reaction Norm Models Improve the Assessment of Genotype by Environment Interaction for Growth, Reproductive, and Visual Score Traits in Nellore Cattle
Source: Animals (Basel). 2022 Sep 29;12(19):2613. doi: 10.3390/ani12192613 (PMC9559514; doi:10.3390/ani12192613)
Supplement: Supplementary file 1 [file animals-12-02613-s001.zip › Supplementary Tables.pdf]

Table S1. Variance estimates and heritability for weaning and yearling traits in Nellore cattle according to the animal model used.

Trait[1]	Model[2]	Variance components[3]				h_a^2 (SE)[3]	AIC
		σ_a^2	σ_m^2	σ_{mpe}^2	σ_e^2		
BWG	AM1	69.73	50.63	60.57	192.15	0.19 (0.01)	3171952.9
	AM2	69.73	50.63	60.57	192.15	0.19 (0.01)	4712265.9
	AM3	55.05	114.09	-	204.72	0.15 (0.00)	3173180.6
	AM4	55.05	114.09	-	204.72	0.15 (0.00)	4713475.7
WC	AM1	0.17	0.10	0.14	0.67	0.16 (0.01)	32116.4
	AM2	0.17	0.10	0.14	0.67	0.16 (0.01)	1572418.2
	AM3	0.14	0.23	-	0.70	0.13 (0.00)	33076.2
	AM4	0.14	0.23	-	0.70	0.13 (0.00)	1573389.2
WP	AM1	0.27	0.07	0.14	0.75	0.22 (0.01)	103284.4
	AM2	0.27	0.07	0.14	0.75	0.22 (0.01)	1643597.3
	AM3	0.24	0.21	-	0.79	0.19 (0.01)	104247.2
	AM4	0.24	0.21	-	0.79	0.19 (0.00)	1644560.2
WM	AM1	0.25	0.08	0.17	0.78	0.19 (0.01)	127413.1
	AM2	0.25	0.08	0.17	0.78	0.19 (0.01)	1667726.0
	AM3	0.21	0.25	-	0.83	0.16 (0.00)	128678.1
	AM4	0.21	0.25	-	0.83	0.16 (0.00)	1668991.1
YW	AM1	334.14	-	-	386.27	0.46 (0.01)	4169122.0
	AM2	334.14	-	-	386.27	0.46 (0.01)	4169103.3
WYG	AM1	91.89	-	-	287.42	0.24 (0.01)	3786419.3
	AM2	91.89	-	-	287.42	0.24 (0.01)	3786400.6
YC	AM1	0.42	-	-	0.68	0.38 (0.00)	1497742.2
	AM2	0.42	-	-	0.68	0.38 (0.00)	1497742.2
YP	AM1	0.42	-	-	0.78	0.35 (0.00)	1548505.4
	AM2	0.42	-	-	0.78	0.35 (0.00)	1548505.4
YM	AM1	0.40	-	-	0.83	0.33 (0.00)	1566460.1
	AM2	0.40	-	-	0.83	0.33 (0.00)	1566460.1
SC	AM1	2.93	-	-	3.88	0.43 (0.01)	2060626.9
	AM2	2.93	-	-	3.88	0.43 (0.01)	2060626.9
AFC	AM1	450	-	-	6593.40	0.06 (0.01)	1606523.3
	AM2	450	-	-	6593.40	0.06 (0.01)	1606523.3

[1] BWG: birth-to-weaning weight gain; WC: conformation at weaning; WP: finishing precocity at weaning; WM: muscling at weaning; YW: yearling weight; WYG: weaning-to-yearling weight gain; YC: conformation at yearling; YP: finishing precocity at yearling; YM: muscling at yearling; SC: scrotal circumference; AFC: age at first calving.

[2] AM1: complete animal model; AM2: complete animal model with intercept; AM3: reduced animal model; AM4: reduced animal model with intercept.

[3] σ_a^2: direct additive genetic variance; σ_m^2: maternal genetic variance; σ_{mpe}^2:maternal permanent environmental variance; σ_e^2: residual variance; h_a^2: direct genetic heritability and standard error.

Table S2. Estimates of variance (diagonals), covariance (upper triangular; in italic) and correlation (lower triangular; in bold) among coefficients of reaction norm models (RNM) for the additive genetic effect of yearling visual score traits in Nellore cattle, with respective residual variance estimates, Akaike information criterion (AIC) and AIC weight (AICw).

Traits[1]	Model[2]	Coefficent[3]	b0	b1	b2	b3	σ^2_e[4]	np[5]	AIC	AICw
YC	RNM_homo	b0 (int)	0.420	*0.006795*			0.67628	4	1497720.7	0.37
		b1 (slp)	**0.40**	0.000693						
	RNM_hete	b0 (int)	0.41969	0.003068			-0.39062	5	1497719.7	0.61
		b1 (slp)	**0.21**	0.000501			0.010162			
	RNM_quad	b0 (int)	0.42242	*0.003992*	*-0.00158*		-0.38822	9	1497729.6	0.00
		b1 (slp)	**0.21**	0.000826	*-0.00033*		0.011917			
		b2 (qdr)	**-0.18**	**-0.87**	0.000177		-0.00323			
	RNM_l-l	b0 (int)	0.4268	*0.008974*	*-0.00964*		-0.38835	9	1497727.5	0.01
		b1 (slp1)	**0.28**	0.002354	*-0.00205*		0.014543			
		b2 (slp2)	**-0.34**	**-0.98**	0.001869		-0.00704			
	RNM_q-q	b0 (int)	0.42217	*0.005997*	*0.000102*	*-0.0028*	-0.38888	14	1497741.7	0.00
		b1 (slp1)	**0.20**	0.002168	*0.000516*	*-0.00161*	0.025113			
		b2 (qdr1)	**0.01**	**0.53**	0.000443	*-0.00081*	0.007897			
		b3 (qrd2)	**-0.10**	**-0.82**	**-0.91**	0.001771	-0.01883			
YP	RNM_homo	b0 (int)	0.42027	0.0051			0.77593	4	1548497	0.00
		b1 (slp)	**0.42**	0.000355						
	RNM_hete	b0 (int)	0.41948	*0.000277*			-0.25297	5	1548486	0.94
		b1 (slp)	**0.03**	0.000159			0.015137			
	RNM_quad	b0 (int)	0.42138	*0.001076*	*-0.00124*		-0.25175	9	1548495	0.01
		b1 (slp)	**0.07**	0.000611	*-0.00043*		0.016778			
		b2 (qdr)	**-0.10**	**-0.95**	0.000339		-0.00223			
	RNM_l-l	b0 (int)	0.42252	*0.003807*	*-0.00521*		-0.24948	9	1548492	0.04
		b1 (slp1)	**0.09**	0.004167	*-0.00504*		0.022849			

		b2 (slp2)	**-0.10**	**-1.00**	0.006117		-0.0115			
	RNM_q-q	b0 (int)	0.42138	*0.001679*	*-0.00076*	*-0.00085*	-0.25235	14	1548500	0.00
		b1 (slp1)	**0.07**	0.001238	*0.000996*	*-0.00171*	0.035013			
		b2 (qdr1)	**-0.03**	**0.67**	0.001804	*-0.0023*	0.011962			
		b3 (qrd2)	**-0.02**	**-0.86**	**-0.96**	0.003216	-0.02459			
YM	RNM_homo	b0 (int)	0.399760	*0.004048*			0.828070	4	1566457	0.06
		b1 (slp)	**0.37**	0.000294						
	RNM_hete	b0 (int)	0.399400	*0.000359*			-0.18850	5	1566451	0.90
		b1 (slp)	**0.03**	0.000300			0.011283			
	RNM_quad	b0 (int)	0.39964	*0.000457*	*-0.00033*		-0.18539	9	1566464	0.00
		b1 (slp)	**0.03**	0.000525	*-0.00028*		0.013520			
		b2 (qdr)	**-0.03**	**-0.7**	0.000309		-0.00402			
	RNM_l-l	b0 (int)	0.401040	*0.002687*	*-0.00383*		-0.18217	9	1566458	0.04
		b1 (slp1)	**0.07**	0.004030	*-0.00558*		0.022773			
		b2 (slp2)	**-0.07**	**-0.97**	0.008265		-0.01866			
	RNM_q-q	b0 (int)	0.399440	*0.007045*	*0.004644*	*-0.00899*	-0.18557	14	1566470	0.00
		b1 (slp1)	**0.3**	0.001350	*0.001237*	*-0.00146*	0.017088			
		b2 (qdr1)	**0.16**	**0.75**	0.002005	*-0.00210*	-0.00166			
		b3 (qrd2)	**-0.3**	**-0.83**	**-0.98**	0.002292	-0.00431			

[1] YC: conformation at yearling; YP: finishing precocity at yearling; YM: muscling at yearling

[2] RNM_homo: linear homoscedastic; RNM_hete: linear heteroscedastic; RNM_quad: quadratic heteroscedastic; RNM_l-l: spline linear–linear heteroscedastic; RNM_q-q: spline quadratic–quadratic heteroscedastic

[3] b0–b3 coefficients of the RNM for the additive genetic random effect [int: intercept; slp: slope; qdr: quadratic; slp1(2): slope segment 1(2); qdr1(2): quadratic segment 1(2)]

[4] Residual variance (RNM_homo) or residual coefficients associated with parameters of heteroscedastic RNM that were modeled using a log-residual function [22].

[5] Number of estimated parameters

Table S3. Average (minimum and maximum) heritability estimates for weaning, yearling and reproductive traits in Nellore cattle for different reaction norm models.

Trait[1]	Models[2]				
	RNM_homo	RNM_hete	RNM_quad	RNM_l-l	RNM_q-q
BWG	0.15 (0.14 - 0.18)	0.15 (0.15 - 0.16)	0.15 (0.15 - 0.27)	0.15 (0.15 - 0.19)	0.15 (0.15 - 0.26)
WC	0.13 (0.11 - 0.16)	0.13 (0.13 - 0.14)	0.14 (0.13 - 0.28)	0.14 (0.13 - 0.19)	0.14 (0.13 - 0.28)
WP	0.19 (0.18 - 0.20)	0.19 (0.19 - 0.20)	0.20 (0.19 - 0.32)	0.19 (0.19 - 0.24)	0.20 (0.19 - 0.31)
WM	0.16 (0.16 - 0.17)	0.16 (0.16 - 0.17)	0.17 (0.16 - 0.29)	0.17 (0.16 - 0.20)	0.17 (0.16 - 0.29)
YW	0.49 (0.34 - 0.70)	0.48 (0.47 - 0.55)	0.49 (0.47 - 0.70)	0.49 (0.47 - 0.63)	0.49 (0.46 - 0.73)
WYG	0.30 (0.08 - 0.64)	0.25 (0.22 - 0.34)	0.30 (0.25 - 0.71)	0.26 (0.24 - 0.42)	0.26 (0.22 - 0.51)
YC	0.38 (0.36 - 0.41)	0.38 (0.38 - 0.39)	0.38 (0.38 - 0.39)	0.38 (0.38 - 0.39)	0.38 (0.38 - 0.39)
YP	0.35 (0.34 - 0.37)	0.35 (0.34 - 0.36)	0.35 (0.34 - 0.38)	0.35 (0.34 - 0.37)	0.35 (0.34 - 0.39)
YM	0.33 (0.31 - 0.34)	0.33 (0.32 - 0.33)	0.33 (0.32 - 0.36)	0.33 (0.32 - 0.35)	0.33 (0.31 - 0.40)
SC	0.45 (0.37 - 0.57)	0.45 (0.41 - 0.52)	0.45 (0.41 - 0.63)	0.45 (0.42- 0.61)	0.45 (0.42 - 0.55)
AFC	0.44 (0.01 - 0.87)	0.12 (0.05 - 0.15)	0.09 (0.06 - 0.64)	0.08 (0.07 - 0.20)	0.09 (0.07 - 0.72)

[1] BWG: birth-to-weaning weight gain; WC: conformation at weaning; WP: finishing precocity at weaning; WM: muscling at weaning; YW: yearling weight; WYG: weaning-to-yearling weight gain; YC: conformation at yearling; YP: finishing precocity at yearling; YM: muscling at yearling; SC: scrotal circumference; AFC: age at first calving.

[2] RNM_homo: linear homoscedastic; RNM_hete: linear heteroscedastic; RNM_quad: quadratic heteroscedastic; RNM_l-l: spline linear–linear heteroscedastic; RNM_q-q: spline quadratic–quadratic heteroscedastic.
