# Supplementary figures and images for: Heteroscedastic Reaction Norm Models Improve the Assessment of Genotype by Environment Interaction for Growth, Reproductive, and Visual Score Traits in Nellore Cattle
Source: Animals (Basel). 2022 Sep 29;12(19):2613. doi: 10.3390/ani12192613 (PMC9559514; doi:10.3390/ani12192613)

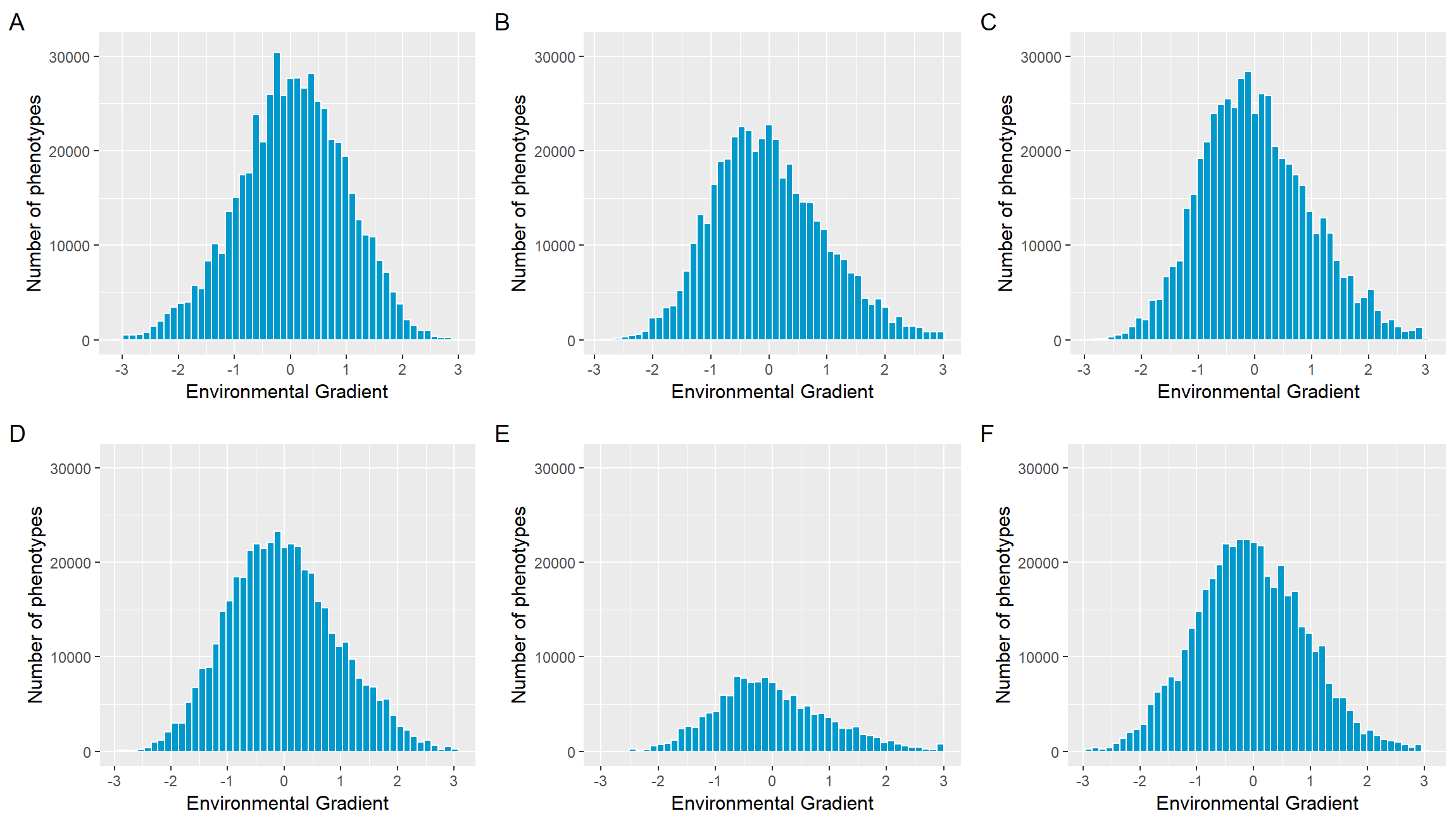

Supplement: Supplementary file 1 [file animals-12-02613-s001.zip › Figures/Figure S1.png]

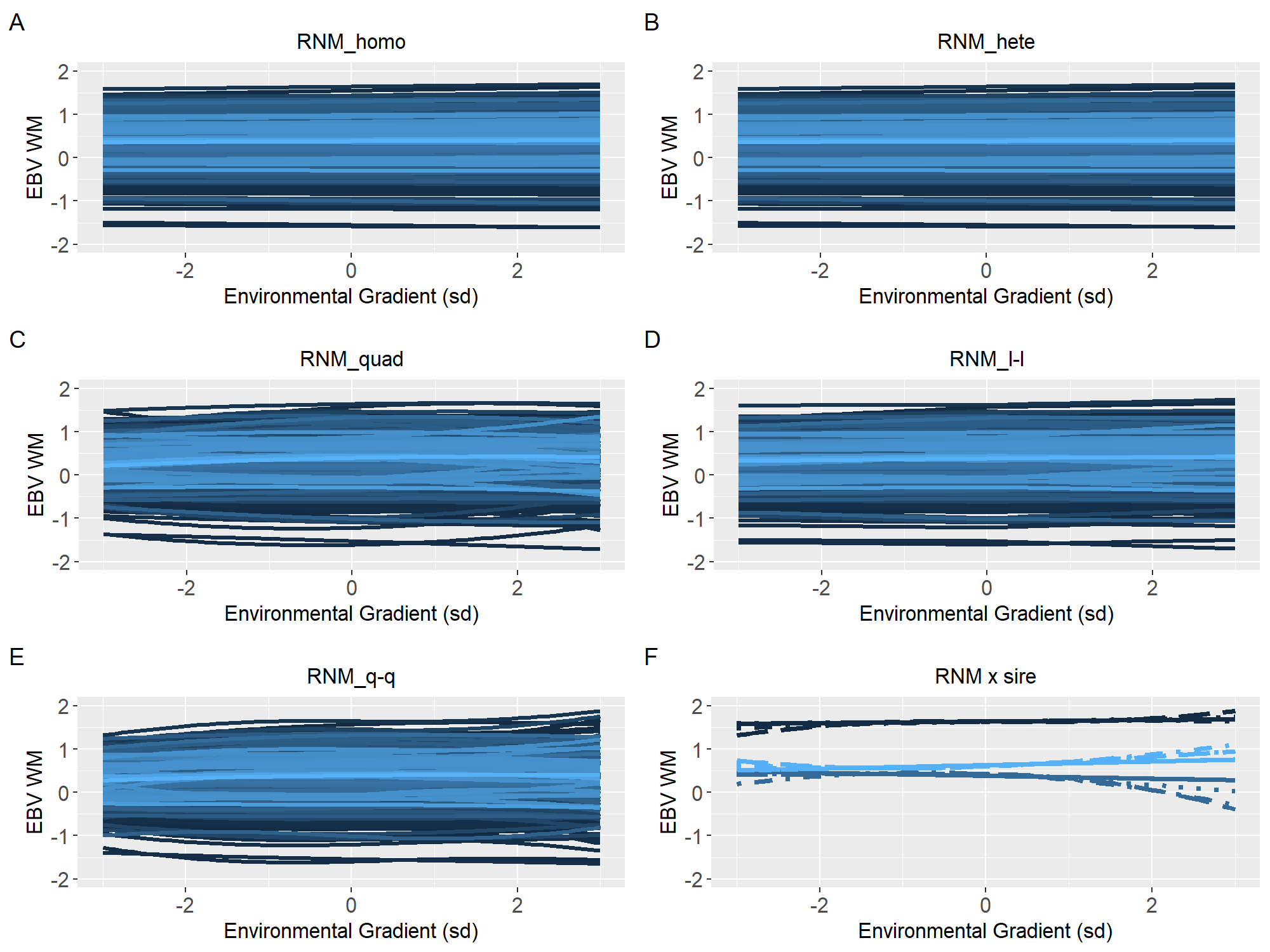

Supplement: Supplementary file 1 [file animals-12-02613-s001.zip › Figures/Figure S10.png]

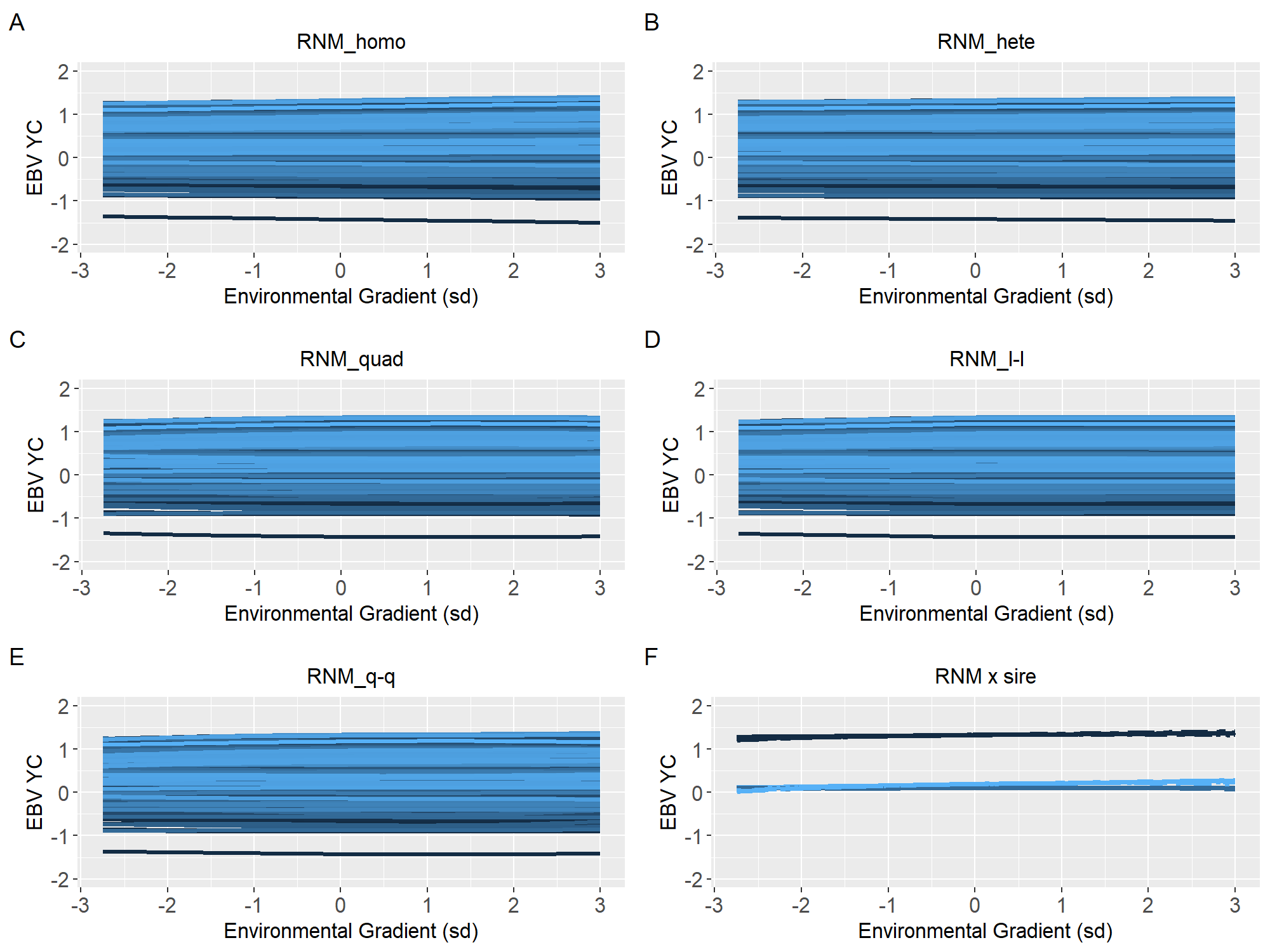

Supplement: Supplementary file 1 [file animals-12-02613-s001.zip › Figures/Figure S11.png]

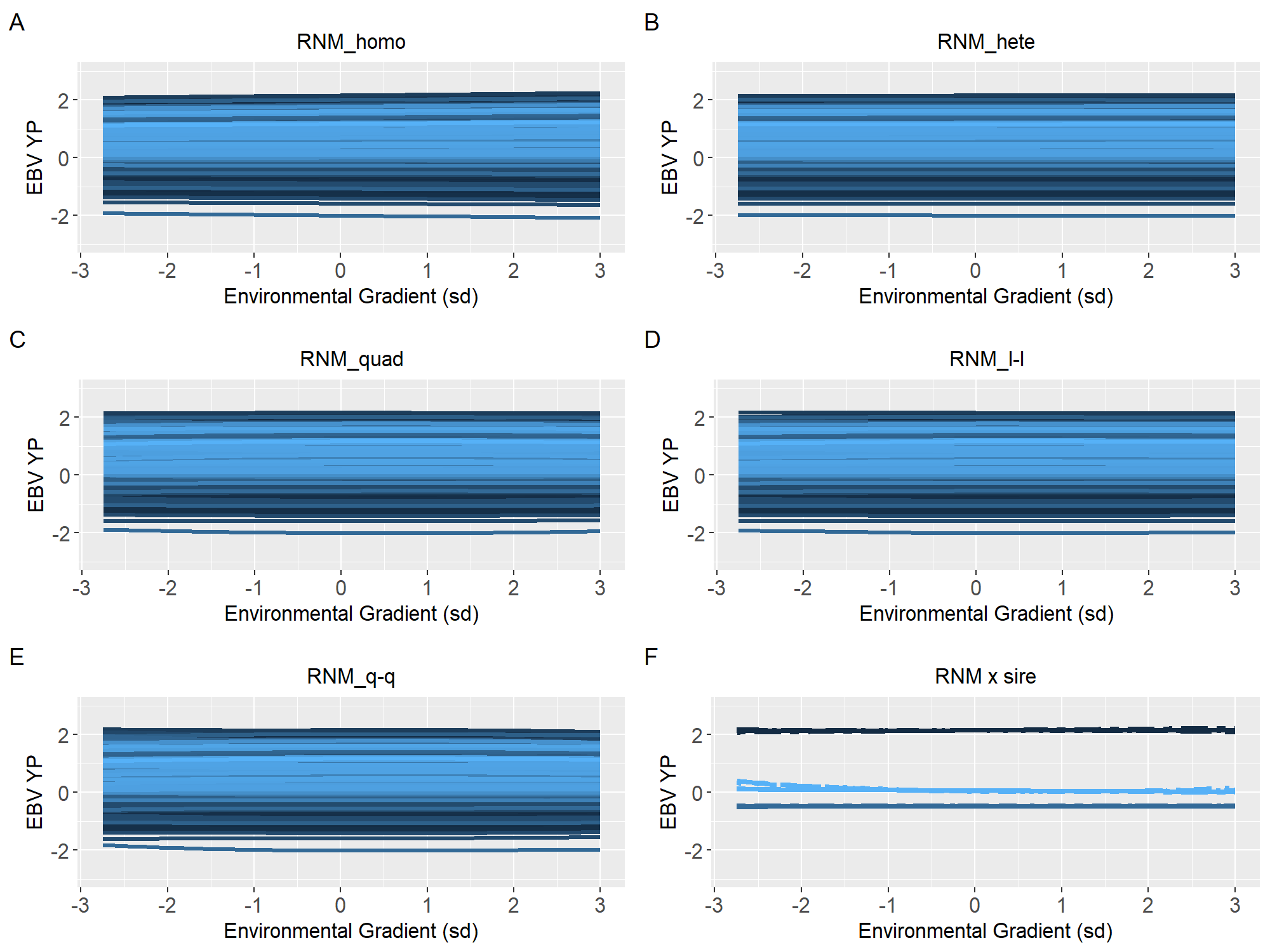

Supplement: Supplementary file 1 [file animals-12-02613-s001.zip › Figures/Figure S12.png]

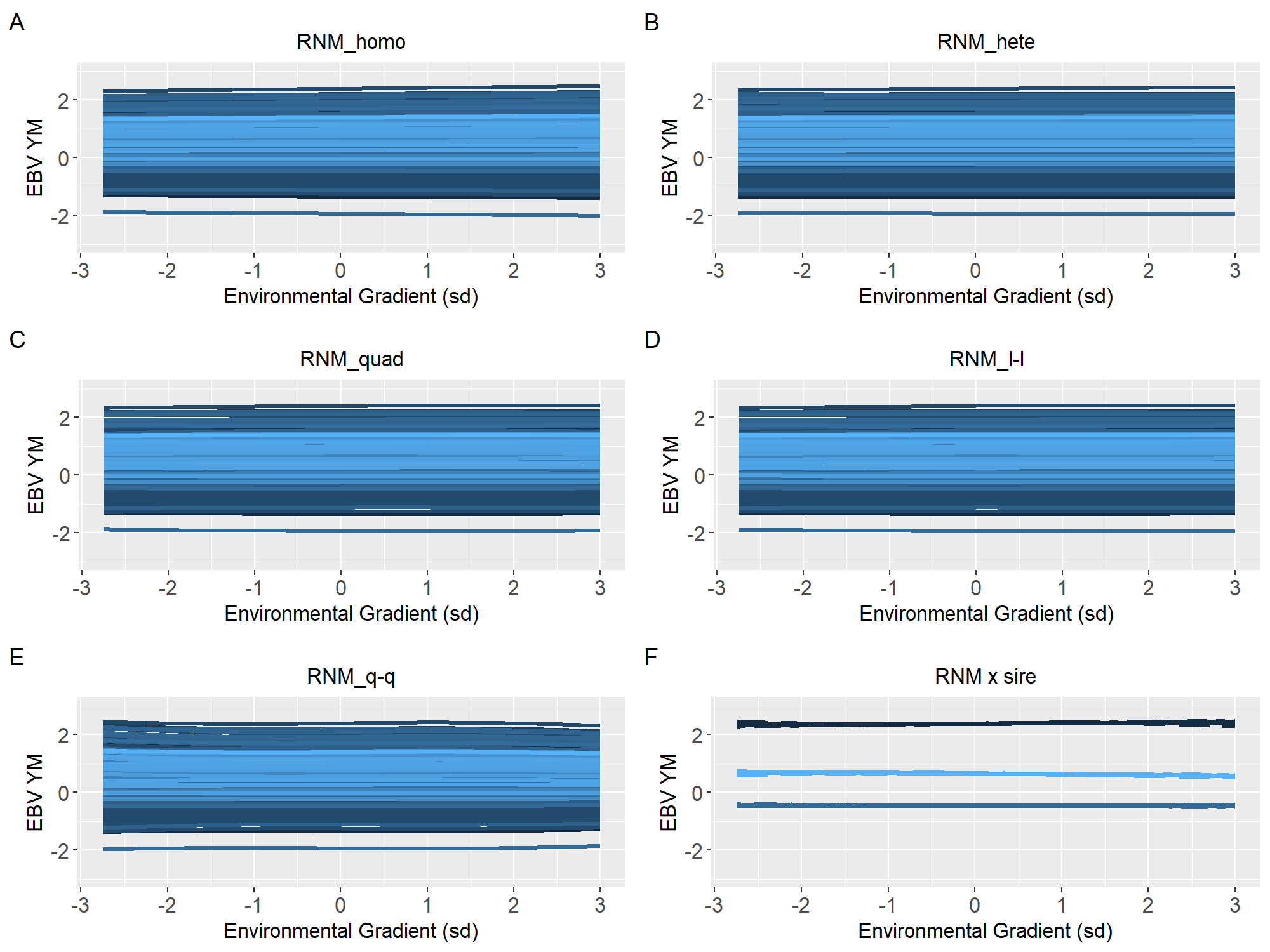

Supplement: Supplementary file 1 [file animals-12-02613-s001.zip › Figures/Figure S13.png]

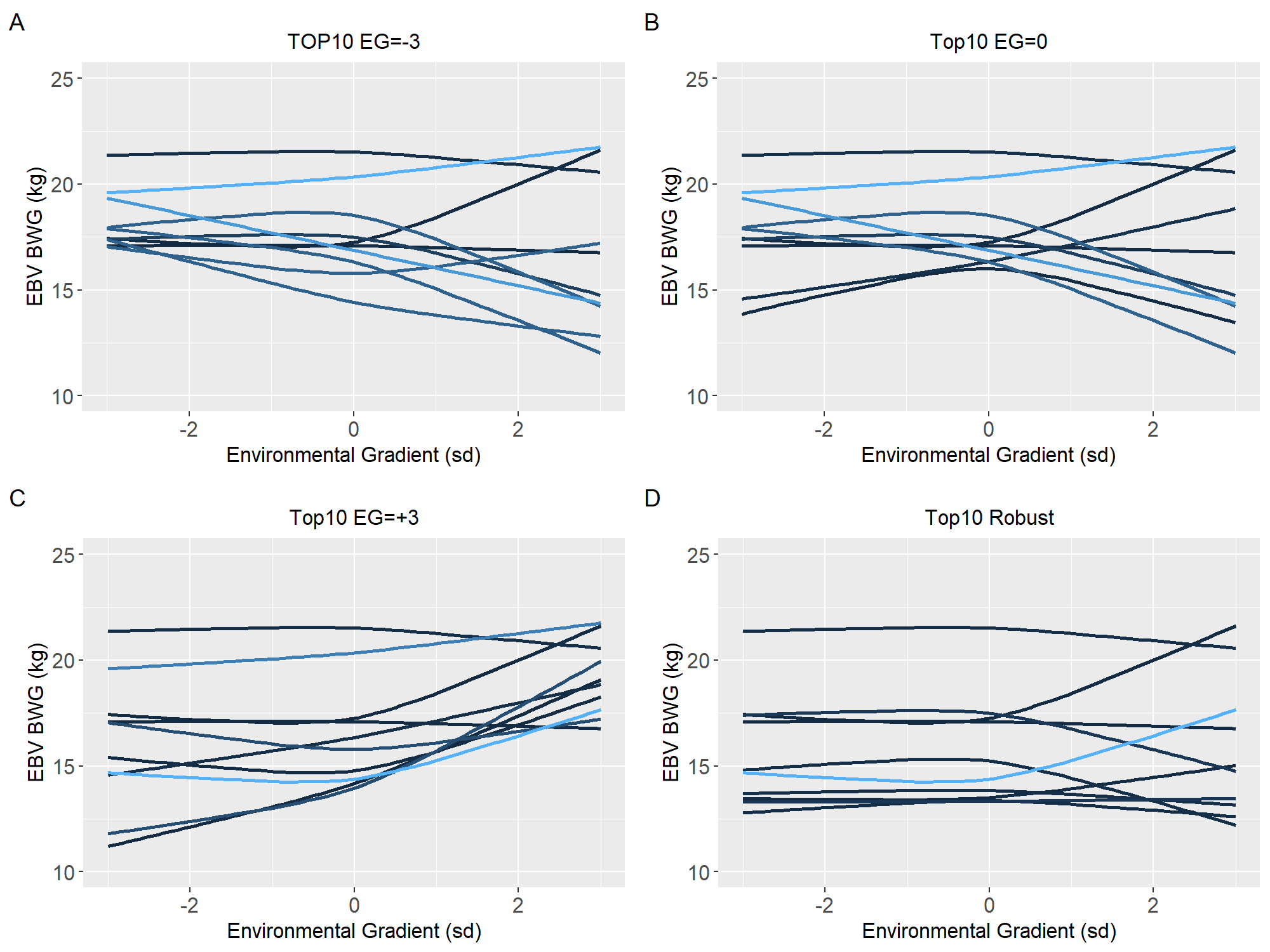

Supplement: Supplementary file 1 [file animals-12-02613-s001.zip › Figures/Figure S14.png]

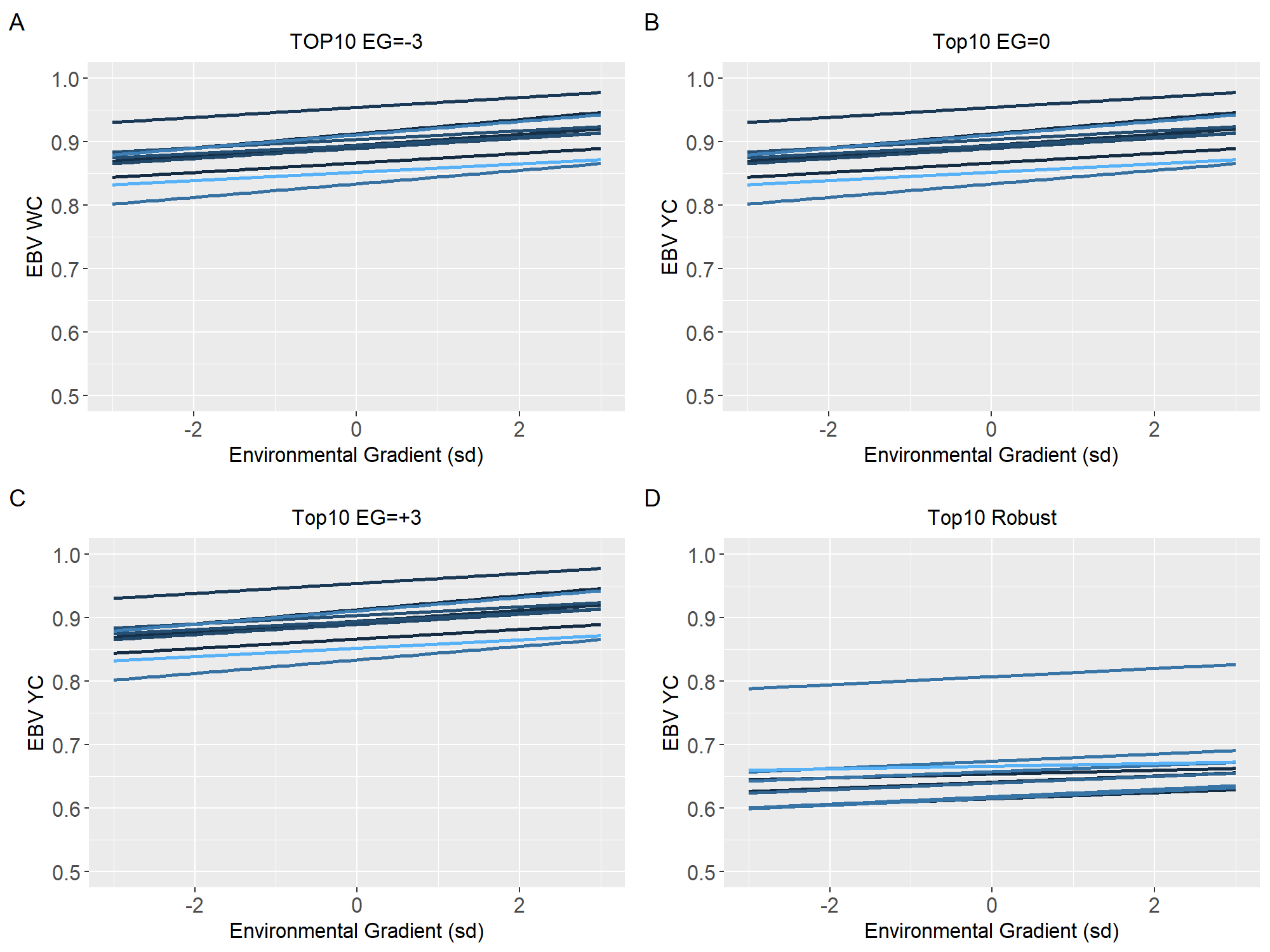

Supplement: Supplementary file 1 [file animals-12-02613-s001.zip › Figures/Figure S15.png]

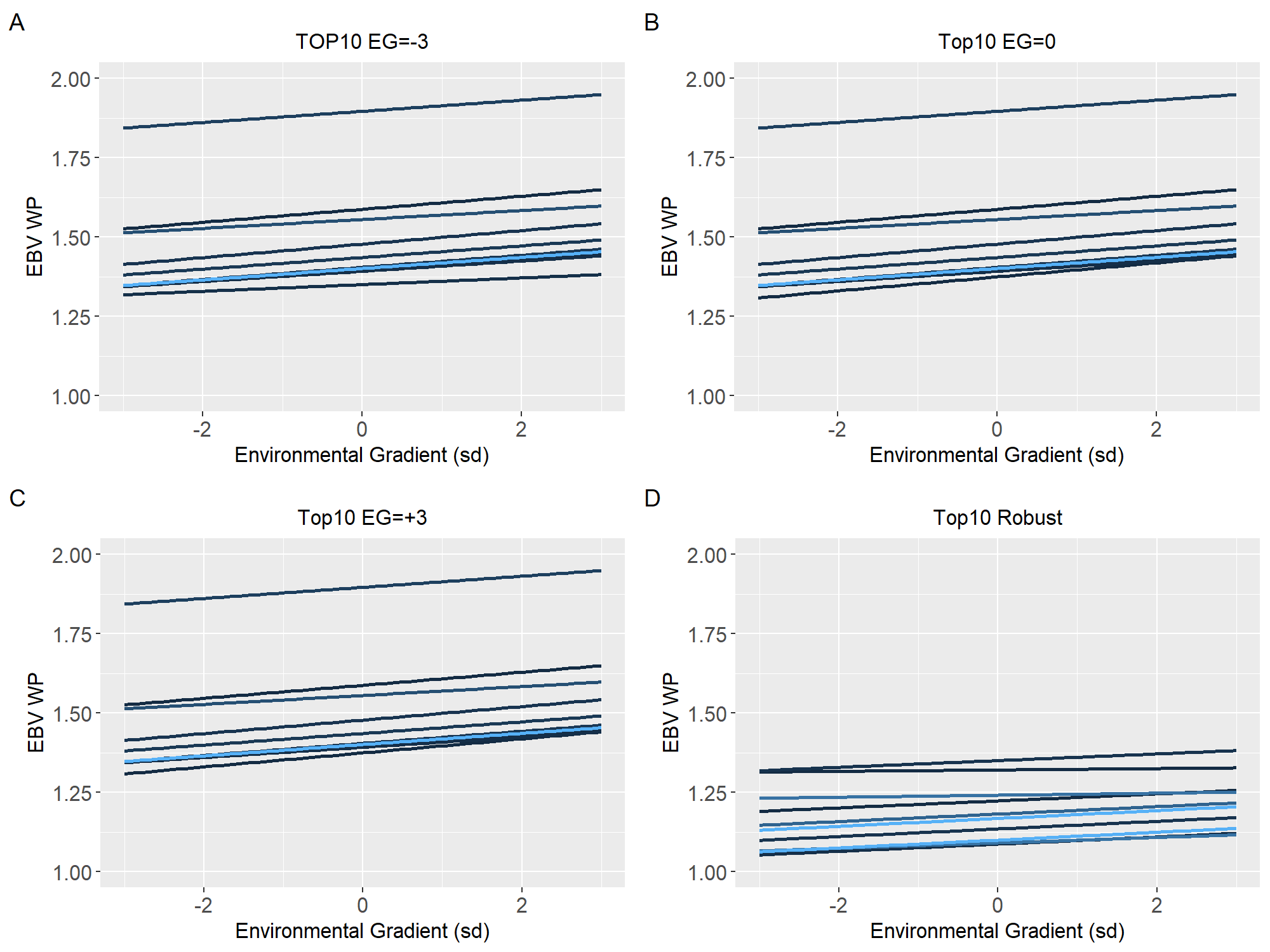

Supplement: Supplementary file 1 [file animals-12-02613-s001.zip › Figures/Figure S16.png]

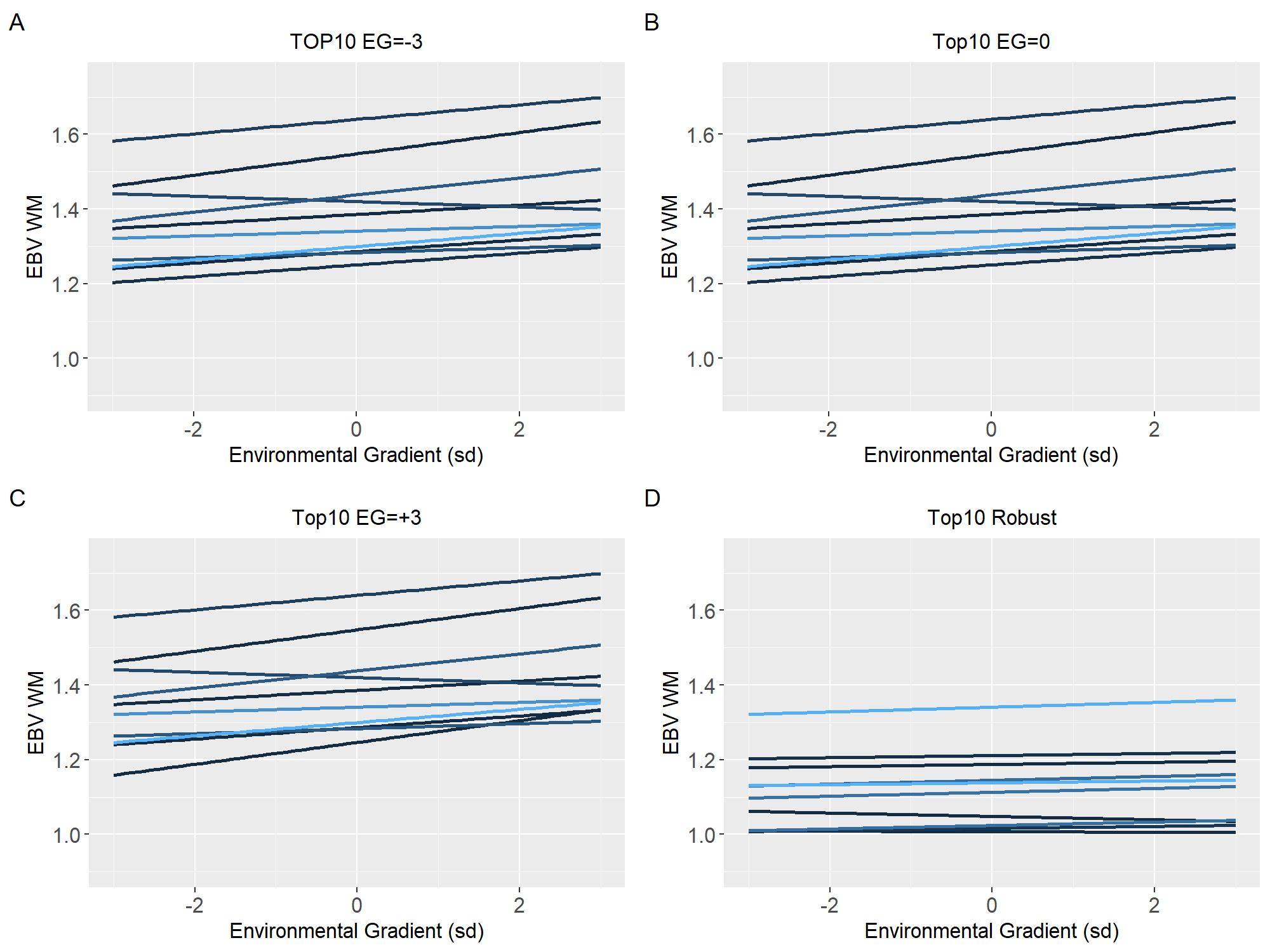

Supplement: Supplementary file 1 [file animals-12-02613-s001.zip › Figures/Figure S17.png]

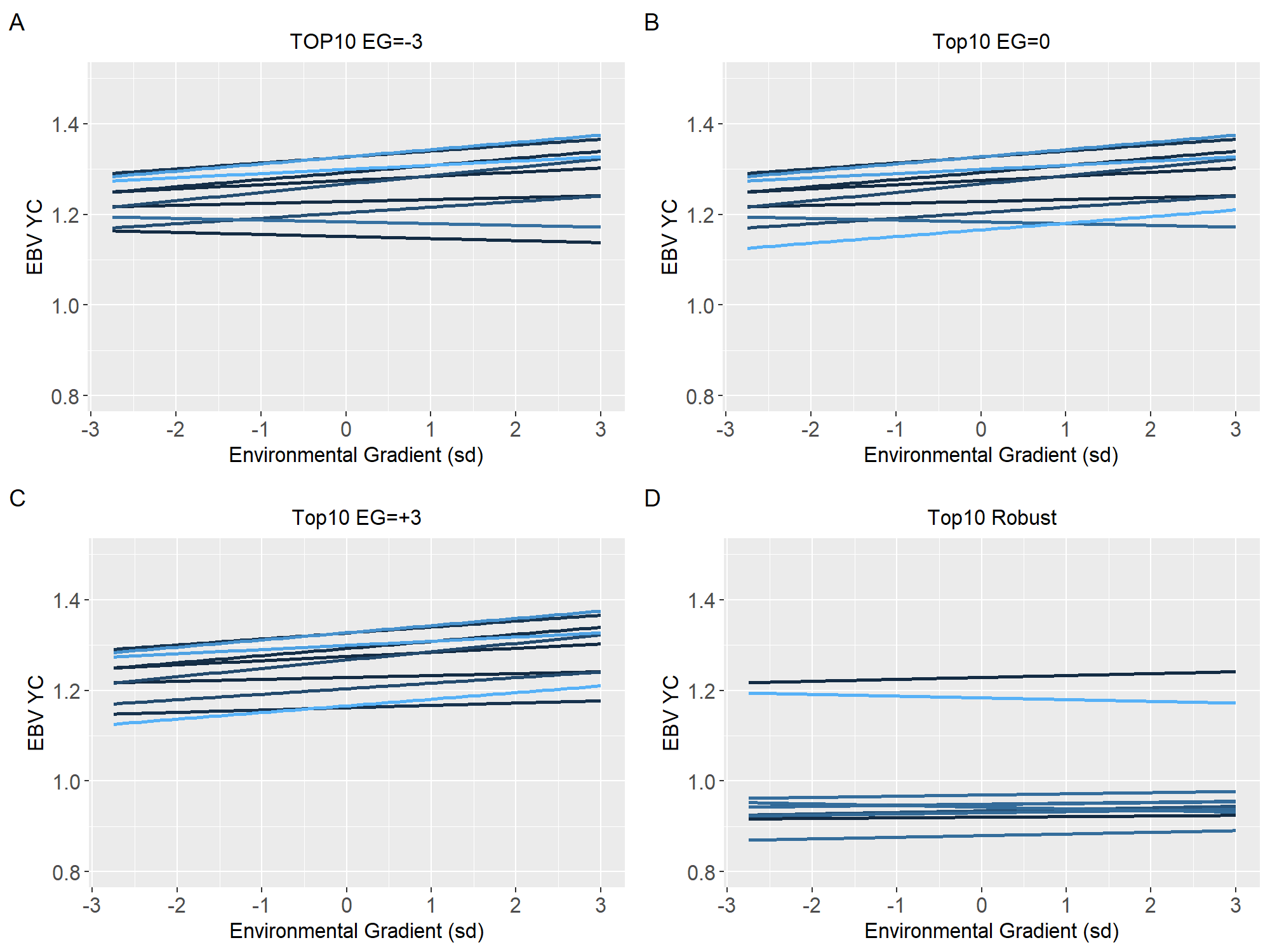

Supplement: Supplementary file 1 [file animals-12-02613-s001.zip › Figures/Figure S18.png]

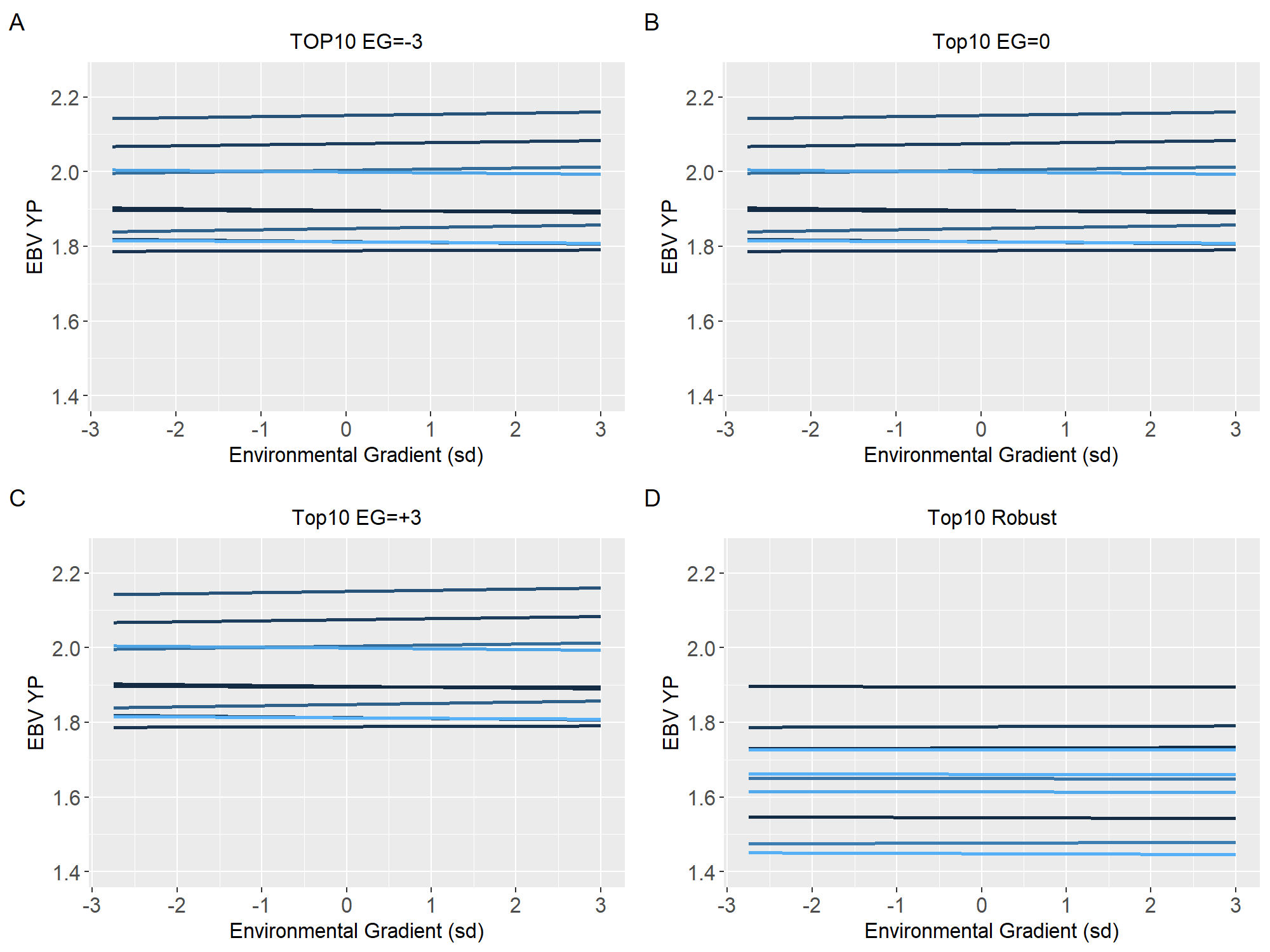

Supplement: Supplementary file 1 [file animals-12-02613-s001.zip › Figures/Figure S19.png]

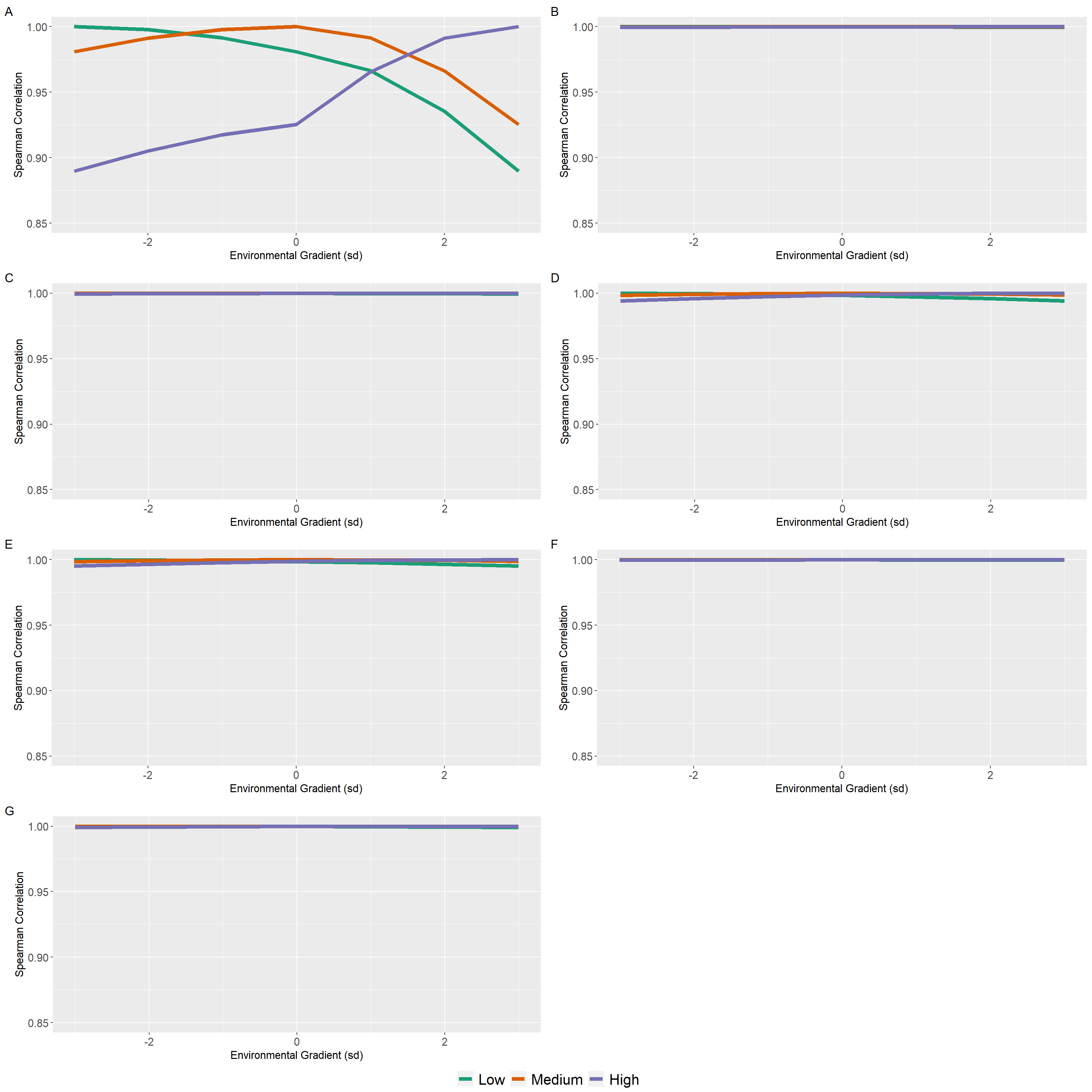

Supplement: Supplementary file 1 [file animals-12-02613-s001.zip › Figures/Figure S2.png]

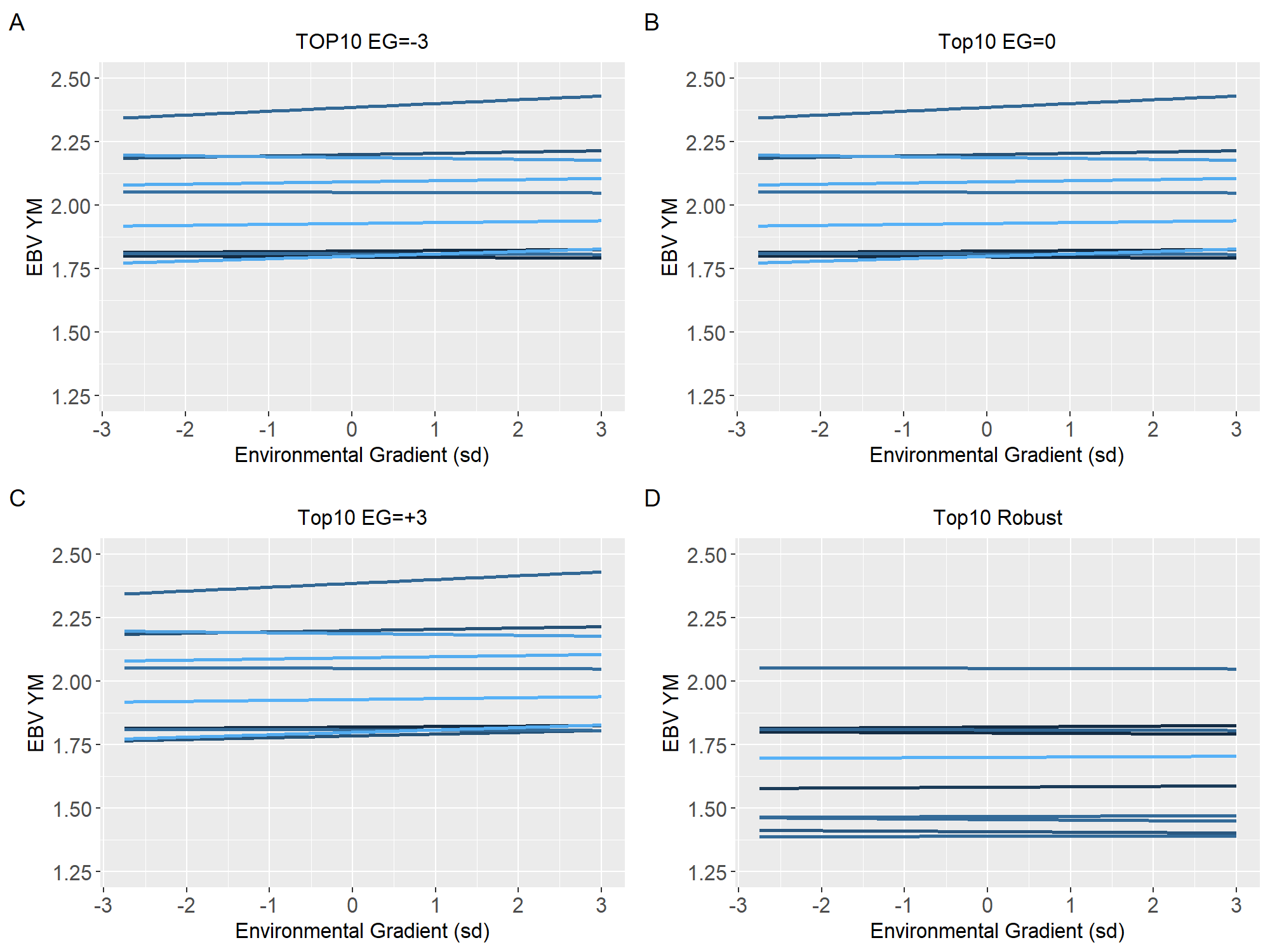

Supplement: Supplementary file 1 [file animals-12-02613-s001.zip › Figures/Figure S20.png]

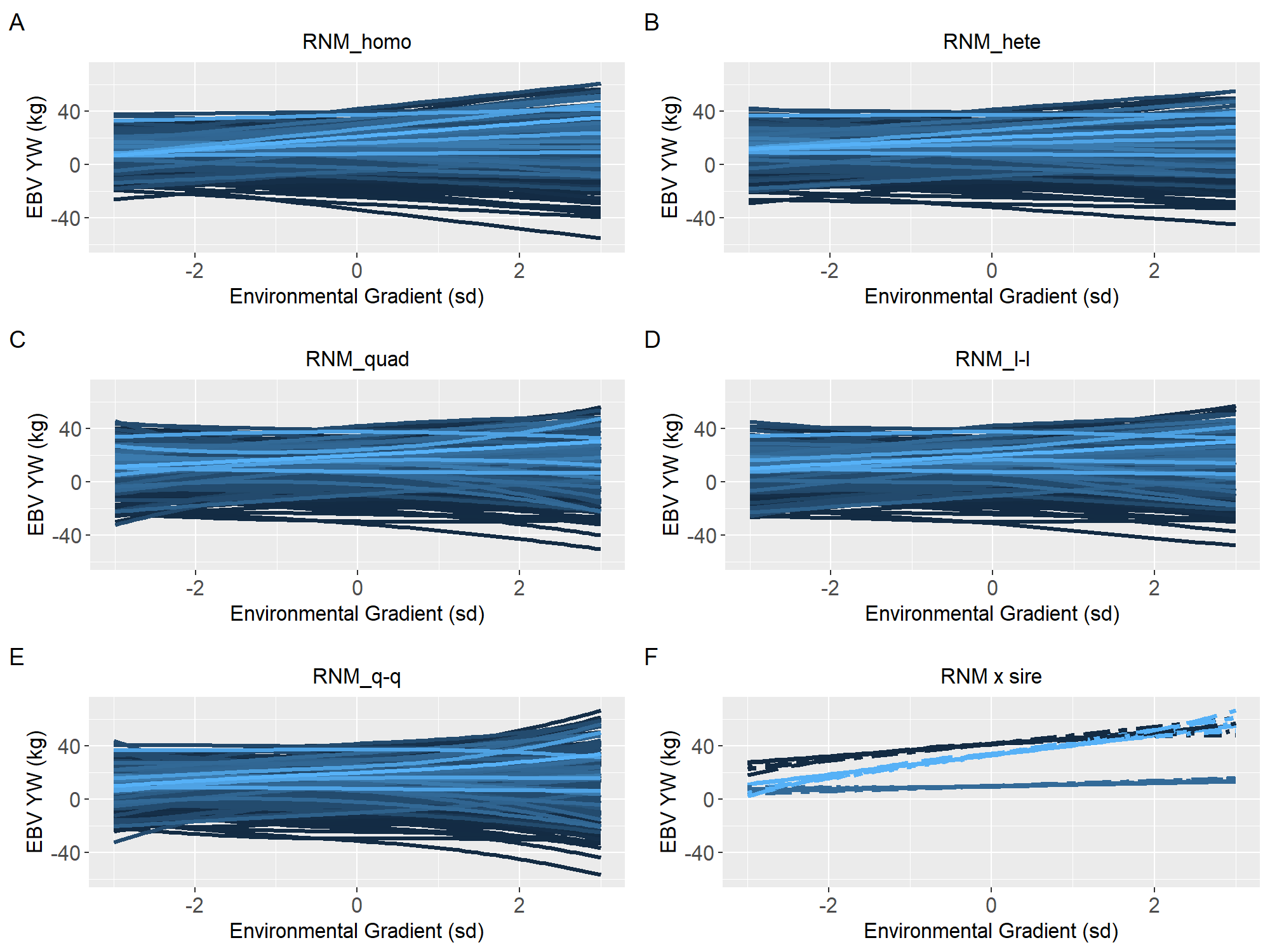

Supplement: Supplementary file 1 [file animals-12-02613-s001.zip › Figures/Figure S3.png]

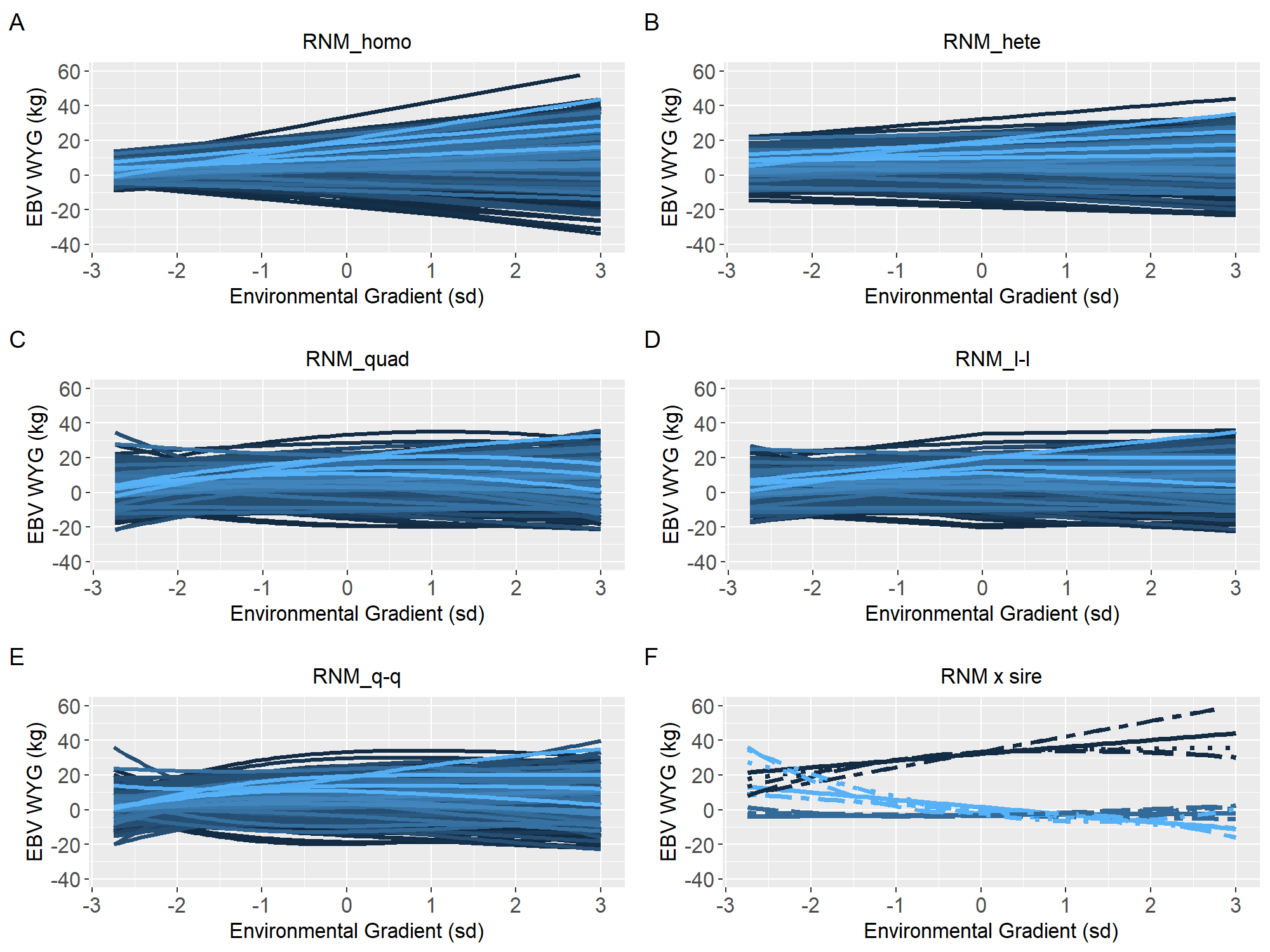

Supplement: Supplementary file 1 [file animals-12-02613-s001.zip › Figures/Figure S4.png]

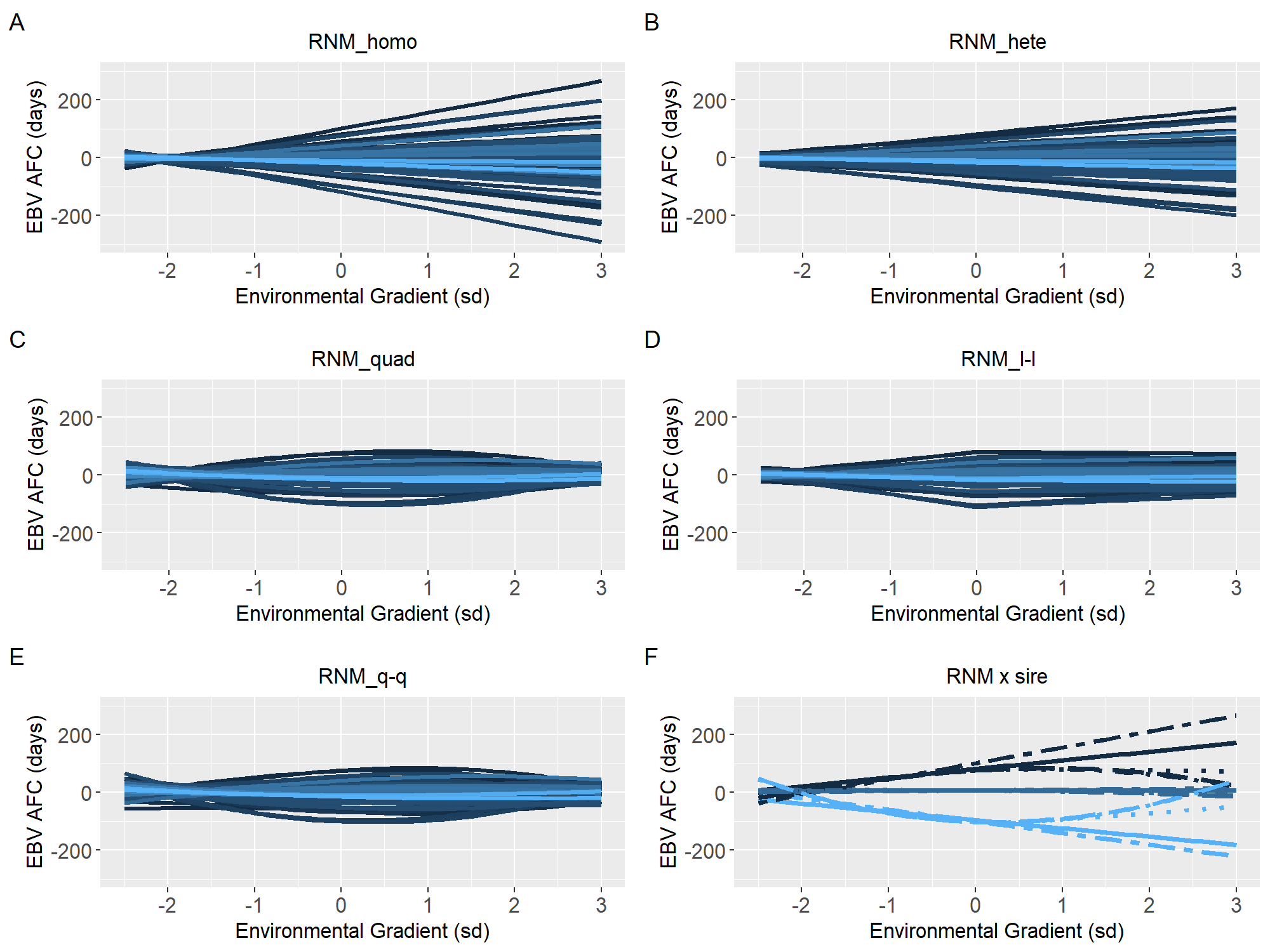

Supplement: Supplementary file 1 [file animals-12-02613-s001.zip › Figures/Figure S5.png]

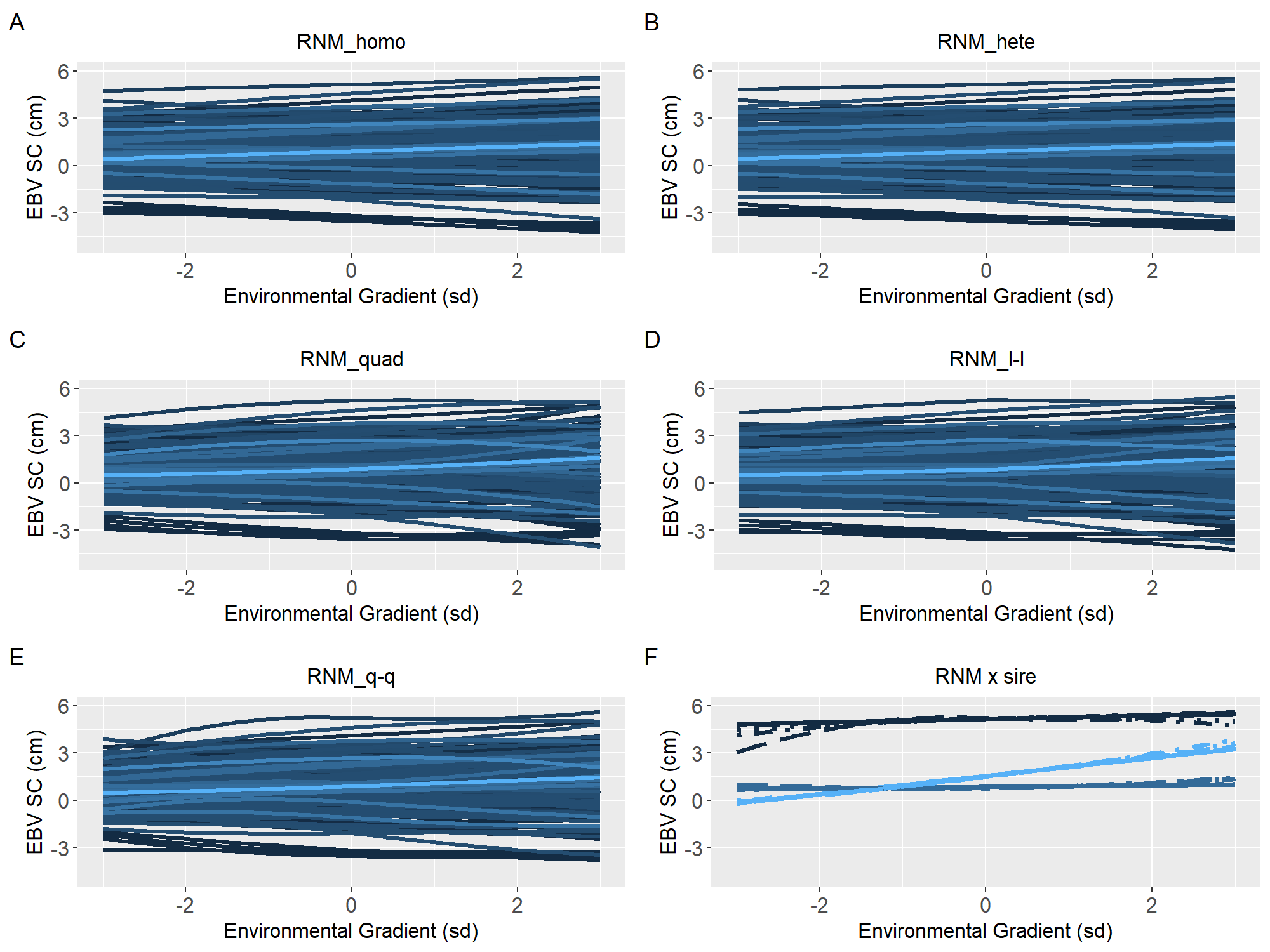

Supplement: Supplementary file 1 [file animals-12-02613-s001.zip › Figures/Figure S6.png]

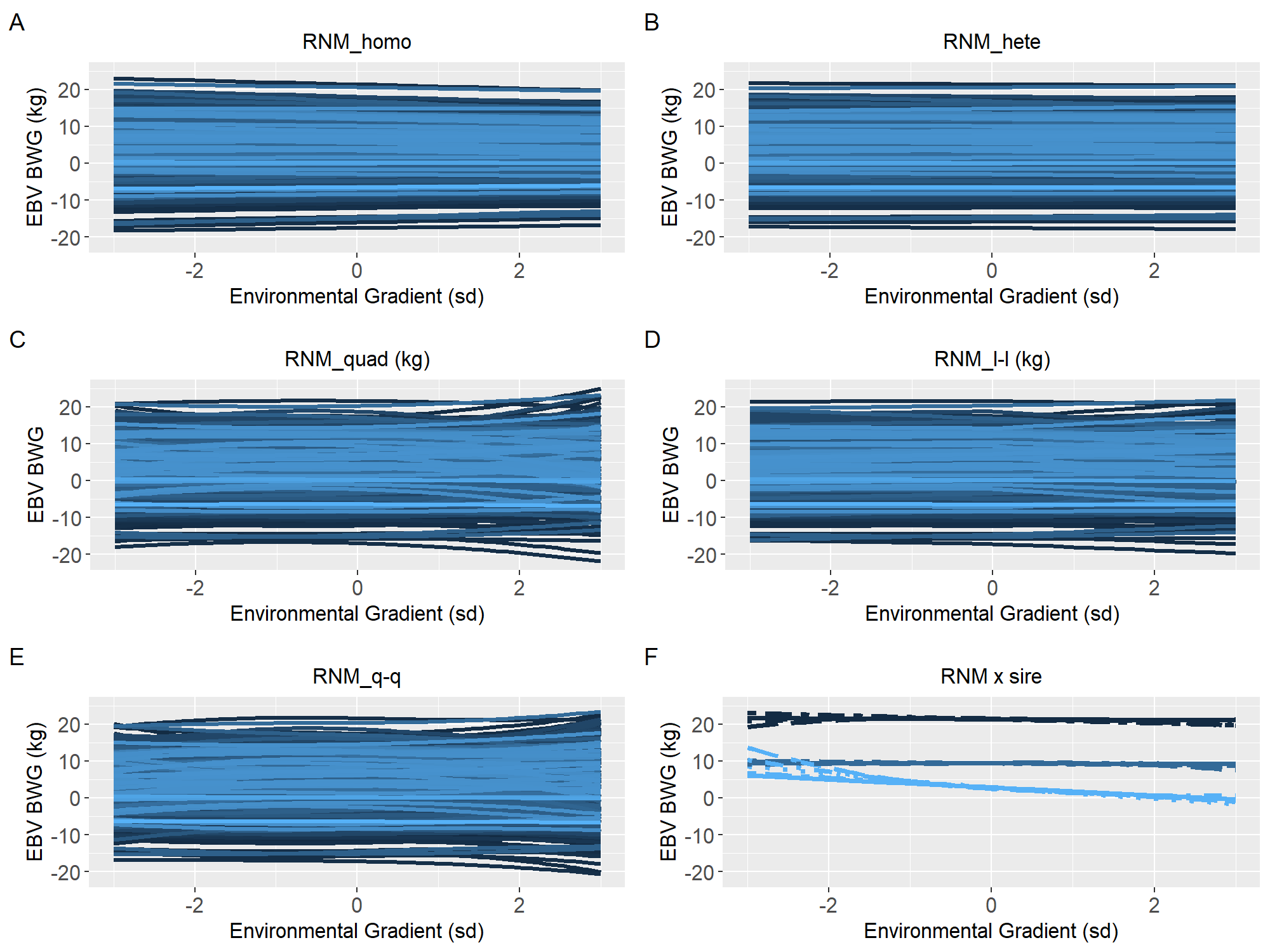

Supplement: Supplementary file 1 [file animals-12-02613-s001.zip › Figures/Figure S7.png]

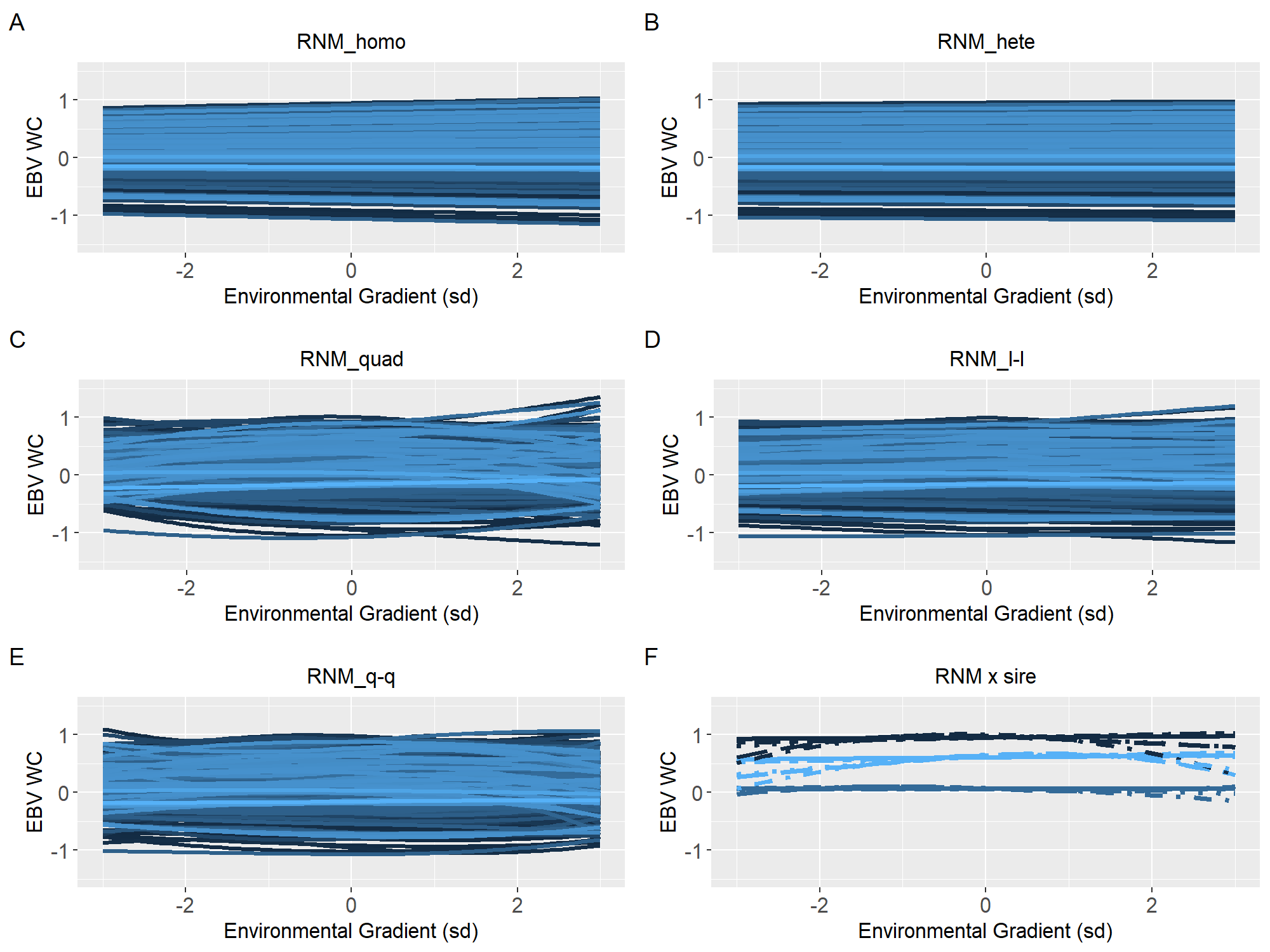

Supplement: Supplementary file 1 [file animals-12-02613-s001.zip › Figures/Figure S8.png]

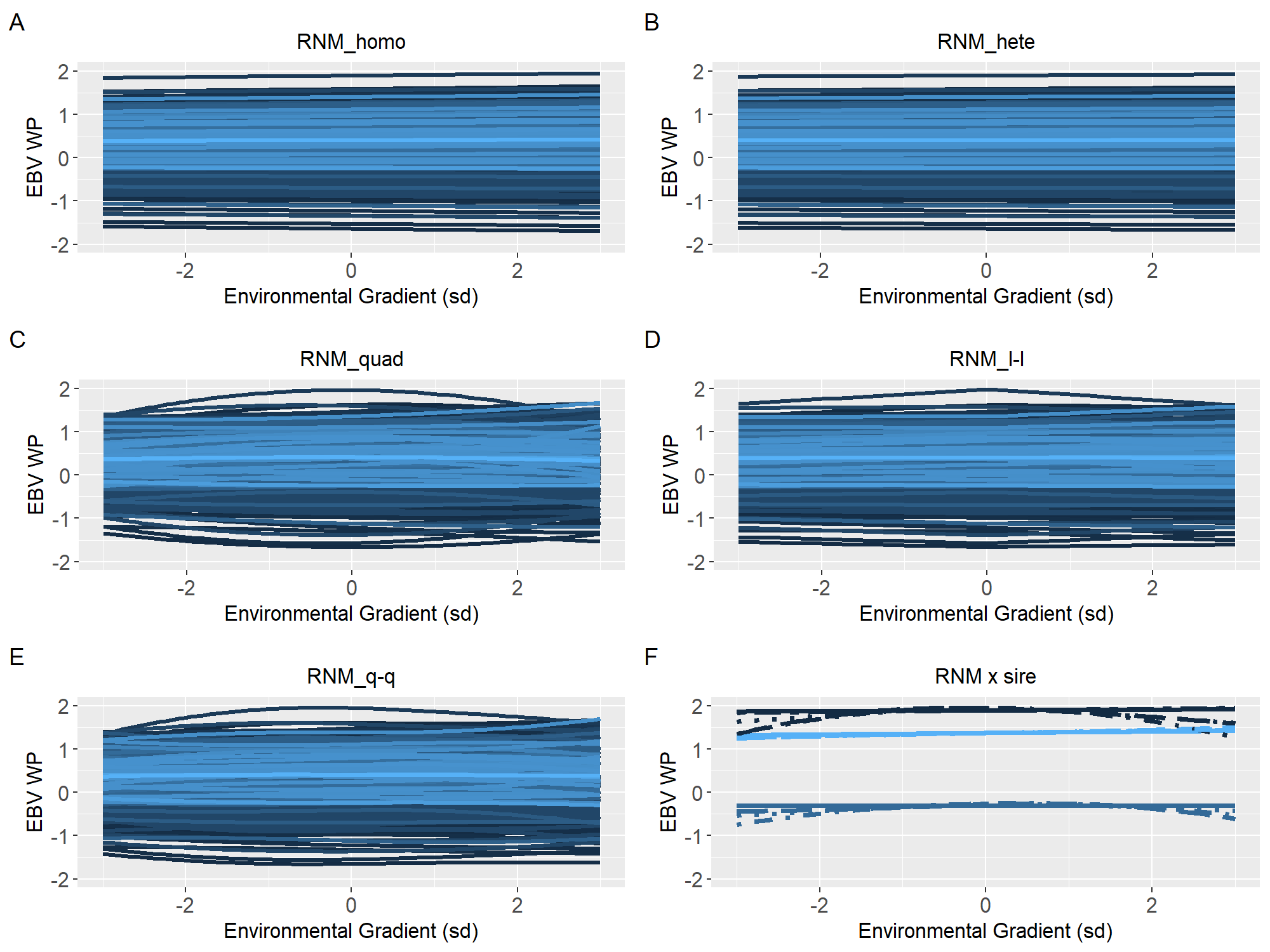

Supplement: Supplementary file 1 [file animals-12-02613-s001.zip › Figures/Figure S9.png]
